# Angiopoietin Like Protein 2 (ANGPTL2) Promotes Adipose Tissue Macrophage and T lymphocyte Accumulation and Leads to Insulin Resistance

**DOI:** 10.1371/journal.pone.0131176

**Published:** 2015-07-01

**Authors:** Yusuke Sasaki, Masayuki Ohta, Dhruv Desai, Jose-Luiz Figueiredo, Mary C. Whelan, Tomohiro Sugano, Masaki Yamabi, Wataru Yano, Tyler Faits, Katsumi Yabusaki, Hengmin Zhang, Andrew K. Mlynarchik, Keisuke Inoue, Ken Mizuno, Masanori Aikawa

**Affiliations:** 1 Center for Interdisciplinary Cardiovascular Sciences, Brigham and Women’s Hospital, Harvard Medical School, Boston, Massachusetts, United States of America; 2 Tokyo New Drug Research Laboratories, Kowa Company, Ltd., Tokyo, Japan; Kurume University School of Medicine, JAPAN

## Abstract

**Objectives:**

Angiopoietin-like protein 2 (ANGPTL2), a recently identified pro-inflammatory cytokine, is mainly secreted from the adipose tissue. This study aimed to explore the role of ANGPTL2 in adipose tissue inflammation and macrophage activation in a mouse model of diabetes.

**Methodology/Principal Findings:**

Adenovirus mediated lacZ (Ad-LacZ) or human ANGPTL2 (Ad-ANGPTL2) was delivered via tail vein in diabetic db/db mice. Ad-ANGPTL2 treatment for 2 weeks impaired both glucose tolerance and insulin sensitivity as compared to Ad-LacZ treatment. Ad-ANGPTL2 treatment significantly induced pro-inflammatory gene expression in white adipose tissue. We also isolated stromal vascular fraction from epididymal fat pad and analyzed adipose tissue macrophage and T lymphocyte populations by flow cytometry. Ad-ANGPTL2 treated mice had more adipose tissue macrophages (F4/80+CD11b+) and a larger M1 macrophage subpopulation (F4/80+CD11b+CD11c+). Moreover, Ad-ANGPTL2 treatment increased a CD8-positive T cell population in adipose tissue, which preceded increased macrophage accumulation. Consistent with our in vivo results, recombinant human ANGPTL2 protein treatment increased mRNA levels of pro-inflammatory gene products and production of TNF-α protein in the human macrophage-like cell line THP-1. Furthermore, Ad-ANGPTL2 treatment induced lipid accumulation and increased fatty acid synthesis, lipid metabolism related gene expression in mouse liver.

**Conclusion:**

ANGPTL2 treatment promotes macrophage accumulation and activation. These results suggest potential mechanisms for insulin resistance.

## Introduction

Obesity, a global burden, closely associates with several health problems such as type 2 diabetes, insulin resistance, atherosclerosis, dyslipidemia, cardiovascular disease, hypertension, and cancer [1, 2]. These diseases increase mortality. Traditional views considered the adipose tissue as a mere energy storage organ. The recent knowledge, however, has recognized the adipose tissue as one of the key organs that regulate systemic metabolism [3–6]. Many macrophages accumulate in adipose tissue in obese individuals and experimental animals. Activated macrophages may induce chronic low-grade inflammation in the adipose tissue, ultimately leading to systemic inflammation and insulin resistance [4–10].

Angiopoietin-like (ANGPTL) proteins are structurally similar to the angiopoietin family proteins which contain both N-terminal coiled-coil domain and C-terminal fibrinogen like domain. Unlike angiopoietins, ANGPTLs do not bind to either the angiopoietin receptor Tie 1 or Tie2, indicating that ANGPTLs have different functions from those of angiopoietin proteins [11]. Some ANGPTLs may regulate metabolism. ANGPTL3 and ANGPTL4 regulate triglyceride metabolism by inhibiting lipoprotein lipase activity [12, 13]. ANGPTL6/Angiopoietin-related growth factor (AGF)-deficient mice develop severe obesity and insulin resistance accompanied by reduced energy expenditure relative to controls [14]. Likewise, ANGPTL2 plays pivotal roles in various inflammatory diseases such as vascular inflammation, obesity, insulin resistance [15], cancer [16, 17] rheumatoid arthritis [18], and atherosclerosis [19, 20]. Elevated serum ANGPTL2 levels positively associate with the development of type 2 diabetes in a general Japanese population [21]. Tabata et al. demonstrated that genetic deletion of ANGPTL2 in mice improved adiposity and systemic insulin resistance [15]. In contrast, Kitazawa *et al*. reported that recombinant ANGPTL2 treatment improved insulin resistance in diabetic db/db mice [22]. Thus, the function of ANGPTL2 on insulin resistance and type 2 diabetes remains unclear. This study aimed to explore the role of ANGPTL2 in adipose tissue inflammation and macrophage activation in vivo.

## Material and Methods

### Reagents

The following antibodies were used in the present study: FLAG M2 HRP antibody (A8692, Sigma Aldrich, St Louis, MO), Human ANGPTL2 monoclonal antibody (Clone 239829, MAB2084, R&D systems Inc., Minneapolis, MN), Beta actin antibody (Clone AC-15, NB600-501, Novus Biologicals, Littleton, CO). We used the following ELISA kits: Human ANGPTL2 Assay ELISA kit (27745, Immuno-Biological Laboratories Co., Ltd, Fujioka, Japan), Human TNF-alpha Quantikine ELISA Kit (DTA00C, R&D systems, Inc., Minneapolis, MN). Phorbol 12-myristate 13-acetate (PMA, P1585-1MG) was purchased from Sigma Aldrich (St Louis, MO).

### Cell cultures

Human monocytoid cell line THP-1 (TIB-202), murine macrophage like cell line RAW264.7 (TIB-71), murine hapatoma cell line Hepa1c1c7 (CRL-2026), murine embryo fibroblast cellline 3T3-L1 (CL-173), murine muscle myoblast cell line C2C12 (CRL-1722), and human hepatocellular carcinoma cell line HepG2 (HB-8065) were purchased from American Type Culture Collection (ATCC, Manassas, VA). The human umbilical vein endothelial cells (HUVECs: CC2517) were purchased from Lonza and maintained in EGM-2 bullet kit (CC-3162, Lonza, Walkersville, MD). THP-1 cells were maintained in RPMI1640 (12-702F, Lonza, Walkersville, MD) supplemented with 10% fetal bovine serum (FBS, Life Technologies, Grand Island, NY), penicillin and streptomycin (Corning, NY) at 37˚C in a humidified atmosphere of 5% CO2. THP-1 monocytes were differentiated by incubation with PMA for 24 h at 37°C. RAW264.7 and Hepa1c1c7 cells were maintained in Dulbecco’s Modified Eagle’s Medium (DMEM, 12-604F, Lonza, Walkersville, MD) supplemented with10% FBS, penicillin and streptomycin at 37˚C in a humidified atmosphere of 5% CO2. 3T3L1 cells were maintained with DMEM supplemented with10% FBS, penicillin and streptomycin at 37˚C in a humidified atmosphere of 5% CO2. For adipocyte differentiation, the cells were grown to confluent for 2 days and then induced with differentiation medium with 1μM dexamethasone (D4902, Sigma Aldrich, St Louis, MO) 0.5 mM isobutylmethylxanthine (IBMX, I-7018, Sigma Aldrich, St Louis, MO), and 10 μg/mL insulin (I-9278, Sigma Aldrich, St Louis, MO). After 2 days, the cells were maintained in DMEM supplemented with10% FBS and 10 μg/mL insulin. Medium was changed every 2 days. C2C12 cells were maintained with DMEM supplemented with10% FBS, penicillin and streptomycin at 37˚C in a humidified atmosphere of 5% CO2. To induce myogenic differentiation, when about 80% cell confluence was attained, the cells were induced with the medium containing 2% horse serum, penicillin and streptomycin. Medium was changed every 2 days. HepG2 cells cells were maintained with DMEM supplemented with10% FBS, penicillin and streptomycin at 37˚C in a humidified atmosphere of 5% CO2. HUVECs were used for each experiment before 6 passages. Human 293f (R790-07) and 293A (R705-07) cells were purchased from Life Technologies (Grand Island, NY) and maintained according to the manufacturer's instructions.

### Construction of adenovirus vector for expression of Angptl2 and production of adenovirus

The adenovirus vector that encodes FLAG-ANGPTL2 was generated by using ViraPower Adenoviral Expression System (K4930-00, Life Technologies, Grand Island, NY).

The coding sequence (CDS) of ANGPTL2 was obtained from Genecopoeia (Cat: GC-U0900, Rockville, MD). To add FLAG tag sequences at C-terminal of ANGPTL2, the following primer set (Forward primer: 5’GACTACAAAGACGATGACGACAAGTAACTCGAGTGCCCGCAACCCAGCTT -3 and Reverse primer: 5’CTTGTCGTCATCGTCTTTGTAGTCGTGGAAGGTGTTGGGGTTCGGTCGG-3) was used and performed FLAG tag insertion by using the site-directed mutagenesis method. FLAG-ANGPTL2 adenovirus expression clone was prepared using pAd/CMV/ V5-DEST Gateway vector (V493-20, Invitrogen, Life Technologies, Grand Island, NY). The recombinant virus (Ad-LacZ or Ad-ANTPGL2) was packaged and amplified in 293A cells according to the manufacturer's instructions. The recombinant virus was packaged and amplified in 293A cells and purified by Fast Trap Adenovirus Purification and Concentration Kit (FTAV00003, EMD Millipore Corporation, Billerica, MA). The virus titer was determined by using Adeno-X Rapid Titer Kit (632250, Clontech Laboratories, Inc., Mountain View, CA).

### Preparation of recombinant human ANGPTL2 protein

Expression and purification method of human ANGPTL2 protein was conducted as followed and slightly modified previous reports [22, 23]. Briefly, C-terminal FLAG ANGPTL2 expression vector was obtained from Origene (Myc-DDK-tagged-Human angiopoietin-like 2, RC201917, Rockville, MD). C-FLAG ANGPTL2 expression vector was transfected with FreeStyle max reagent (16447–100, Life Technologies, Grand Island, NY) into 293f cells. After 72 hour, conditioned medium was collected and passed through a 0.2 μm pore size filter (4612, PALL Life Sciences, Ann Arbor, MI). ANTI-FLAG M2 Affinity Gel (A2220, Sigma Aldrich, St Louis, MO) was added into the filtered conditioned media. FLAG M2 Gel was washed with Tris-Buffered Saline (TBS) and elute with 165 mg/mL FLAG peptide (F3290, Sigma Aldrich, St Louis, MO) or Gly-HCl (pH3.5) buffer. For Gly-HCl elution, we immediately added 10 x TBS to neutralize the solution. Protein concentration was determined by BCA protein assay kit (23225, Thermo Scientific, Rockford, MA). Protease inhibitor cocktail (P8340, Sigma Aldrich, St Louis, MO) was added throughout the procedures.

### Mouse study design

All experiments were conducted using 12-wk-old male mice. We obtained 10-wk-old C57BL6J mice and diabetic db/db mice from Jackson Laboratories (Bar Harbor, ME). The mice were maintained on a 12-h light, 12-h dark cycle in normal cages with food and water ad libitum. Lean mice or diabetic db/db were received 5 x10^9 plaque forming units (p.f.u.) of adenovirus solution (Ad-LacZ or Ad-ANGPTL2) via tail vein at 12 week old. We monitored body weight on Day 0 (Adenovirus Injection), 3, 7, 10 and 14. Food intake and blood glucose level were monitored once a week. Study design was described in S1 Fig. Mouse studies were conducted in accordance with federal guidelines. The Institutional Animal Care and Use Committee (Beth Israel Deaconess Medical Center, Boston, protocol #041–2012) approved all study protocols.

### Glucose and insulin tolerance tests

Glucose tolerance test was performed on Day 7 or Day14 after adenovirus injection. Mice were fasted overnight then given glucose (1 g/kg body weight) by oral administration. For insulin tolerance test, mice were fasted 4 hr then given insulin solution (2 Unit/kg, Novolin, Novo Nordisk, Plainsboro Township, NJ) via i.p. injection. The sterilized mouse tail was snipped using a scalpel and gently squeezed to obtain tail blood. Blood glucose levels were measured using glucometer (One Touch Ultra, Lifescan Inc., Milpitas, CA) at indicated time points (time 0, 15, 30, 60 and 120) after glucose or insulin administration.

### Stromal vascular cell isolation and flow cytometry analysis

Isolation of stromal vascular fraction (SVF) from mice epididymal fat pad was performed as previously described [24, 25]. Epididymal fat pads of db/db mice were weighed, rinsed 3 times with phosphate buffered saline (PBS), and then minced in the MACS buffer (PBS with bovine serum albumin, EDTA, and 0.09% Azide, 130-091-221, Miltenyi Biotec Inc., San Diego, CA). Tissue suspensions were incubated with 1 mg/mL collagenase Type II (C6885, Sigma Aldrich, St Louis, MO) and 0.2 mg/mL DNase I (DN25, Sigma Aldrich, St Louis, MO) at 37°C for 30 min. with shaking (90 rpm). Cell suspension were filtered through 100 μm cell strainer (REF352360, BD Bioscience, San Jose, CA) and then centrifuged at 200 x g for 5 min to separate floating adipocytes from the SVF pellets. The SVF pellets were incubated with RBC lysis buffer (420301, Biolegend, San Diego, CA) for 5 min before centrifuge (300 x g, 5 min) and resuspended in MACS buffer. Stromal vascular fraction cells were incubated with Fc Block (Clone2.4G2, 553142 BD Biosciences, San Jose, CA) for 20 min at 4°C. Cells were stained with the following conjugated antibodies (30 min, 4°C in the dark): FITC anti-mouse F4/80, PE/Cy5 anti-mouse CD11b, APC/Cy7 anti-mouse CD11c, PE anti-mouse CD3, FITC anti-mouse CD4, and APC anti-mouse CD8a (all were purchased from Biolegend, San Diego, CA). After staining, cells were gently washed twice and resuspended in the fresh MACS buffer. Stained cells were analyzed by FACS (BD FACS Aria II, BD Biosciences, San Jose, CA). Adipose tissue macrophages and pro-inflammatory M1 macrophages were identified as F4/80+ CD11b+ cells, and F4/80+ CD11b+ CD11c+, respectively.

### Analyses of lipid profile, liver triglyceride, insulin and adipokine levels

Plasma total cholesterol and triglyceride levels were measured by Cholesterol E-test kit (Wako, Japan), and LabAssay Triglyceride test (Wako, Japan), respectively. The liver triglyceride assay was performed as previously described [26]. 200 mg portion of the liver was extracted with 10 ml of chloroform–methanol 2:1 in a Polytron (Kinematica GmbH, Luzern, Switzerland). A 0.2 ml aliquot of the lower phase was taken, evaporated and dissolved in isopropanol. Liver cholesterol and triglyceride levels were measured by Cholesterol E-test kit (Wako, Japan), and LabAssay Triglyceride test (Wako, Japan), respectively. Plasma adiponectin and insulin levels were evaluated by Mouse Adiponectin/Acrp30 Quantikine ELISA Kit (MRP300, R&D systems, Minneapolis, MN), and Rat/Mouse Insulin ELISA (EZRMI-13K, EMD Millipore Corporation, Billerica, MA), respectively.

### Histological analyses

Epididymal fat pads were fixed with 4% paraformaldehyde and embedded in paraffin. Tissues were sectioned serially (6 μm) and stained with hematoxylin and eosin (H&E) for general morphology. Liver samples were embedded in OCT compound (Sakura Finetek U.S.A., Inc, Torrance, CA). We built software tool using OpenCV (open-source image analysis library, Garage Willow, Menlopark, CA) to be able to calculate the adipocyte size accurately. Cross sectional images of adipose tissues were processed through several image filters consisting of Canny edge filter, binarizing filter and labeling filter to extract each adipocyte in the images. The adipocyte areas obtained as pixel numbers were converted to physical area in um^2. For oil Red O staining, slides were rinsed with distilled water, and placed in absolute propylene glycol for 2 min. Then, slides were stained with 5 mg/mL oil red O (3125–12, EMD Millipore Corporation, Billerica, MA) solution for 90 min. After staining, slides were differentiated with 85% propylene glycol solution and rinse with distilled water. Slides were stained in Gill’s hematoxylin solution. After rinse with distilled water for several time and apply aqueous mounting solution and covered with cover glass. Stained area was quantified with Hue Saturation Value Analysis Program as described previously [27].

### Gene expression analysis

Total RNA extracted from cells with TRIzol (Life technologies, Grand Island, NY) underwent cDNA synthesis with High-Capacity cDNA Reverse Transcription Kit (Life technologies, Grand Island, NY). Real-time quantitative PCR employed TaqMan gene expression assays (Life technologies, Grand Island, NY) and ABI 7900HT sequence detection system (Life Technologies, Grand Island, NY). TaqMan probes which used in this study were summarized in the S1 Table (mouse) and S2 Table (human).

### Akt phosphorylation assay

Adenovirus which expressed with LacZ or ANGPTL2 was infected to differentiated 3T3L1 adipocytes (on Day7) differentiated C2C12 myotubes (on Day4), and HepG2 when the cells reached confluence. For adenovirus transduction in 3T3L1 adipocytes, we used density-based separation followed by re-plating of enriched adipocytes in monolayer (DREAM) method previously reported [28]. Adenovirus particles were infected with AdenoMag (AM71000, OZ bioscience, San Diego, CA) kit according to the manufacturer's instructions. The cells were applied serum starvation overnight and treated with medium with insulin solution (10, 100, and 1000 nM) for 15 min. The cells were then washed with ice-cold phosphate buffered saline (PBS) twice, and added lysis buffer (9803, Cell Signaling Technology, Danvers, MA) with protease inhibitor and Phostop phosphatase inhibitor (Roche, Indianapolis, IN). For western blotting analysis, Proteins were resolved by 10% sodium dodecyl sulfate-polyacrylamide gel electrophoresis (SDS-PAGE) and electrotransferred to nitrocellulose membranes (Life Technologies, Grand Island, NY). After blocking with 2% non-fat milk, the membranes were probed with antibodies for pSer473-PKB and total PKB (4060S and 4691S, Cell Signaling Technology, MA, USA) and the proteins were visualized by SuperSignal West Pico Chemiluminescent Substrate (34087, Thermo Fisher Scientific Inc., Rockford, IL). For phospho and total Akt ELISA, PathScan Phospho-Akt1 (Ser473) and Total Akt1 Sandwich ELISA Antibody Pair (#7143 and #7142, Cell Signaling Technology, Danvers, MA) according to the manufacturer's instructions.

### Statistical analysis

Statistical analysis was performed with GraphPad Prism 5 (GraphPad Software, La Jolla, CA). The data were analyzed using unpaired t-test or Mann-Whitney test for two group comparison. Differences involving multiple groups were first tested by one-way ANOVA, followed by post-hoc analysis with Dunnett’s test adjusting for multiple comparison when comparing multiple groups to a control group. Differences with p < 0.05 were considered statistically significant. All data are presented as mean ± standard error of mean (SEM).

## Results

### ANGPTL2 impaired glucose tolerance and insulin sensitivity in db/db mice

**Fig 1:**
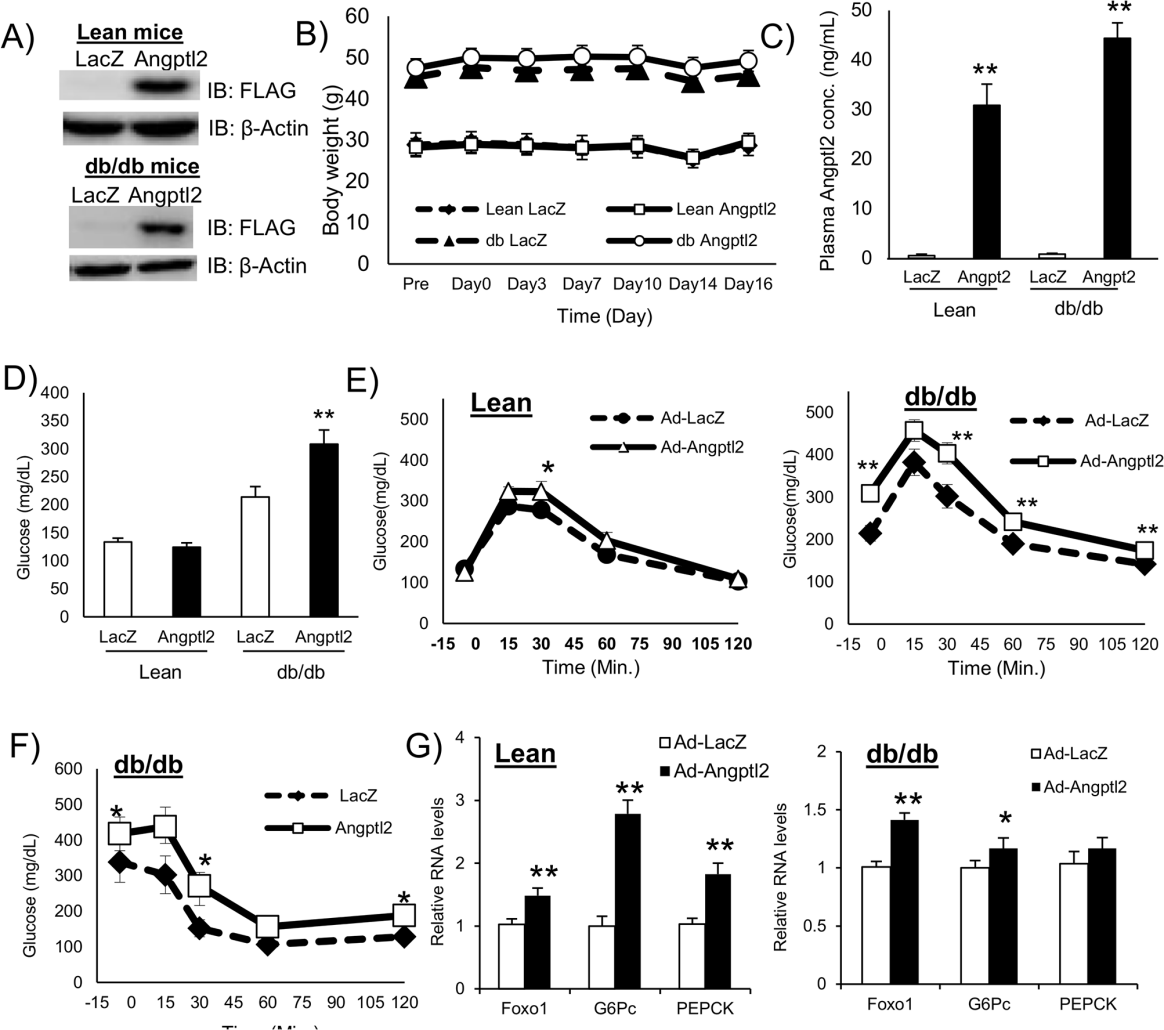
Enforced expression of human ANGPTL2 impaired glucose and insulin tolerance in mice. A, Western blot analysis of FLAG-tagged ANGPTL2 in the liver (upper panel: lean mice, lower panel: db/db mice). B, Body weight (n = 7–8 mice / group). C, Plasma ANGPTL2 levels 2 week after adenovirus injection (n = 6–8). D, Fasting glucose levels at 2 weeks (n = 7–8 mice / group). E, Glucose tolerance test at 2 weeks after treatment (Left: Lean mice, Right: db/db mice, n = 7–8 mice / group, in Experiment 1). F, Insulin tolerance test 2 weeks after treatment in db/db mice (n = 7–8 mice / group, in Experiment 2). G, Quantitative RT-PCR of mRNAs encoding gluconeogenesis related genes in the liver (Left: Lean mice, Right: db/db mice, n = 8). Data are mean ± SEM, **: P<0.01, *: P<0.05 compared with LacZ group.

We prepared adenovirus particles expressing FLAG fusion human ANGPTL2 protein (Ad-ANGPTL2) for in vivo gain-of-function experiments. We used db/db mice, a diabetic mutant strain, as a model of spontaneous type 2 diabetes. We injected Ad-ANGTL2 in db/db mice via tail vein. FLAG-ANGPTL2 protein expression was confirmed in the liver on Day14 (Fig 1A). Ad-ANGPTL2 treatment did not affect body weight (Fig 1B and S1B Fig). Ad-ANGPTL2 treatment increased circulating ANGPTL2 levels in both lean and db/db mice (Fig 1C and S1C Fig). Fasting glucose level increased in Ad-ANGPTL2 treated db/db mice on Day 14, however, there was no difference in lean mice (Fig 1D). There was no significant difference on plasma insulin, adiponectin, total cholesterol (except for lean mice), and triglyceride levels (S1D, S1E, S1F and S1G Fig). Furthermore, enforced expression of ANGPTL2 had minimal effects on food intake (S1H Fig). We performed glucose tolerance test on Day14. Ad-ANGPTL2 treatment impaired glucose tolerance in both lean and db/db mice (S1E Fig). We also performed insulin tolerance test using db/db mice on Day 14 (S1A Fig, experiment 2). Two week of Ad-ANGPTL2 treatment exacerbated insulin sensitivity (Fig 1F). After tissue harvesting, gluconeogenesis related gene expression was analyzed. Angptl2 overexpression enhanced Foxo1, G6Pc and Pepck expression in both lean and obese mice (Fig 1F). These results indicate that Angptl2 overexpression worsen both glucose tolerance and insulin sensitivity.

### ANGPTL2 induced pro-inflammatory gene Expression in adipose tissue

**Fig 2:**
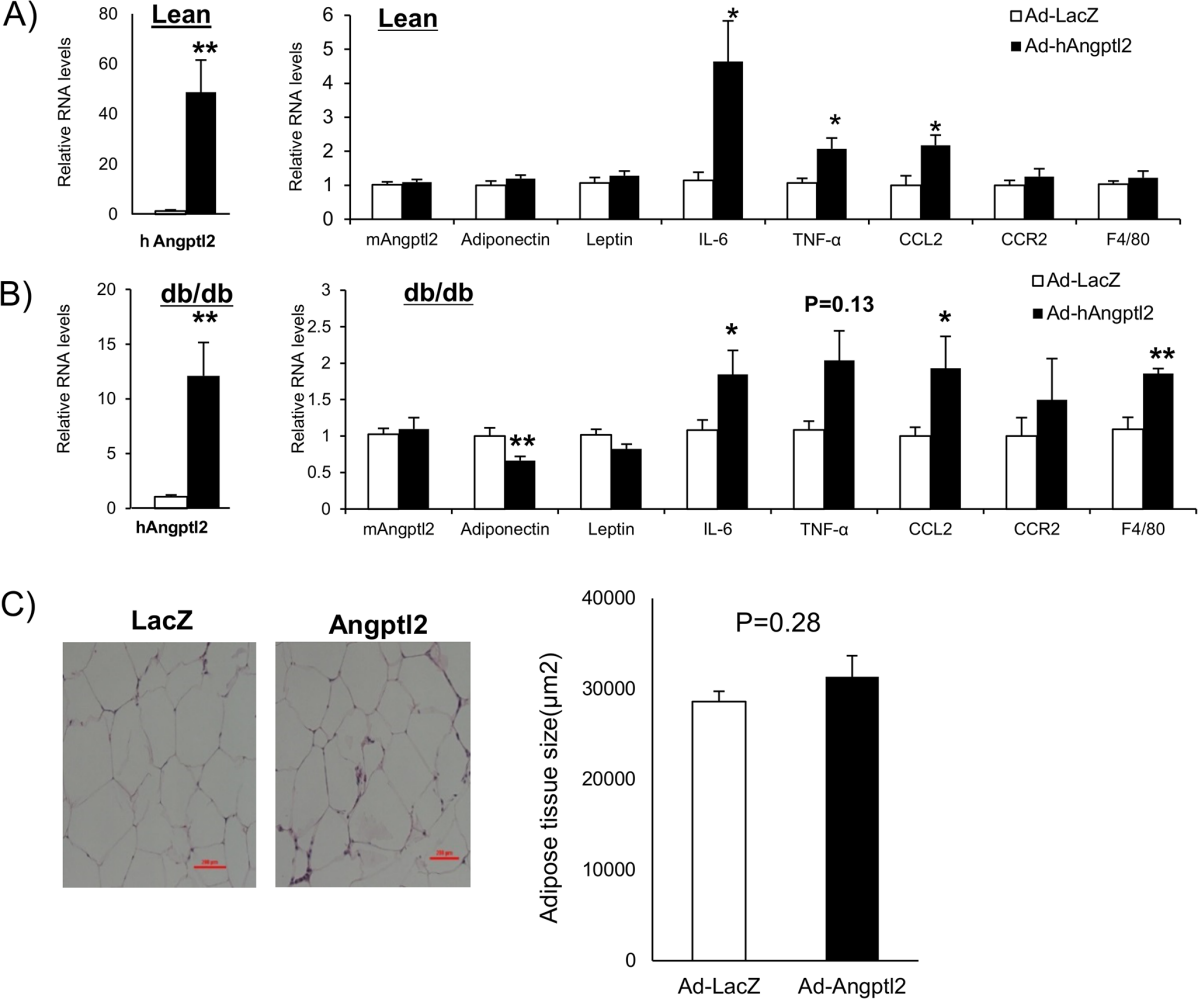
Enforced expression of human ANGPTL2 induced inflammation in adipose tissue. A, Quantitative RT-PCR of mRNAs encoding adipokines and pro-inflammatory genes in epididymal adipose tissue (lean mice, n = 8) B, Quantitative RT-PCR of mRNAs encoding adipokines and pro-inflammatory genes in epididymal adipose tissue (db/db mice, n = 5–8). C, Representative images of adipocytes after H&E staining and quantification of adipocyte size in epididymal fat (db/db mice, n = 7, Scale bar: 200 μm). Data are mean ± SEM, **: P<0.01, *: P<0.05 compared with LacZ group.

We then assessed the effects of enforced expression of human ANGPTL2 in mouse adipose tissue. Real time PCR analysis showed that Ad-ANGPTL2 treatment enhanced human ANGPTL2 expression in both lean and db/db mice, while endogenous mouse Angptl2 expression did not change (Fig 2A and 2B). ANGPTL2 increased pro-inflammatory gene expression such as IL-6, TNF-α, and CCL2 (Fig 2A and 2B). Moreover, ANGPTL2 overexpression reduced adiponectin expression. F4/80 gene expression, indicative of macrophage burden, increased only in db/db mice. Induction of F4/80 gene expression after Ad-ANGPTL2 treatment may imply the increase of adipose tissue macrophages. Histological analysis of epididymal fat pad in db/db mice indicated no effects of Ad-ANGPTL2 treatment on adipocyte size (Fig 2C).

### ANGPTL2 increased adipose tissue macrophages and pro-inflammatory M1 macrophages

**Fig 3:**
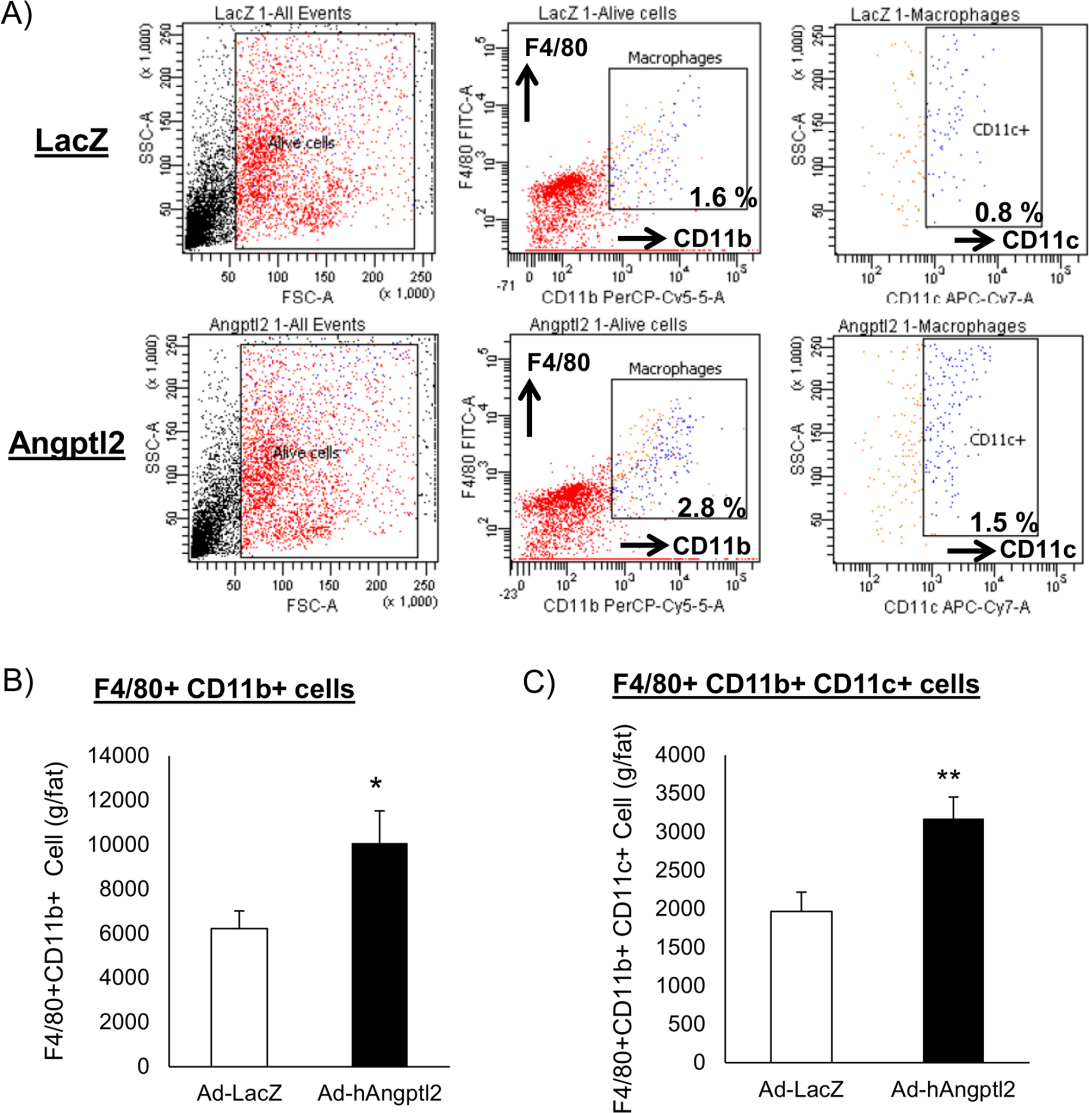
Enforced expression of human ANGPTL2 increased adipose tissue macrophages and promoted M1 macrophage polarization in adipose tissue from db/db mice. A, Stromal vascular fraction (SVF) was isolated from the epididymal fat pad then stained with F4/80, CD11b, and CD11c antibodies and analyzed by FACS. B, Adipose tissue macrophage number was determined as F4/80+ and CD11b+ fraction and determined by FACS (n = 7–8). Data are mean ± SEM, **: P<0.01, *: P<0.05 compared with LacZ group.

Our findings in Fig 2 indicate that ANGPTL2 may increase macrophage accumulation and pro-inflammatory responses in the adipose tissue. To further address this hypothesis, we stained stromal vascular fractions isolated from epididymal fat pad with F4/80, CD11b and CD11c antibodies. We quantified F4/80 and CD11b double-positive macrophages and pro-inflammatory M1 macrophages (F4/80+CD11b+CD11c+) by fluorescence-activated cell sorting (FACS) analysis. Ad-ANGPTL2 treatment increased both adipose tissue macrophages (F4/80+CD11b+) and the pro-inflammatory M1 macrophage population (F4/80+CD11b+CD11c+) after 2-week treatment (Fig 3A, 3B and 3C), which agreed with gene expression profile (Fig 2A and 2B). The results indicate that ANGPTL2 may induce chronic inflammation in adipose tissue.

### Increased accumulation of CD8 positive T cells by ANGPTL2 preceded macrophage accumulation in adipose tissue

**Fig 4:**
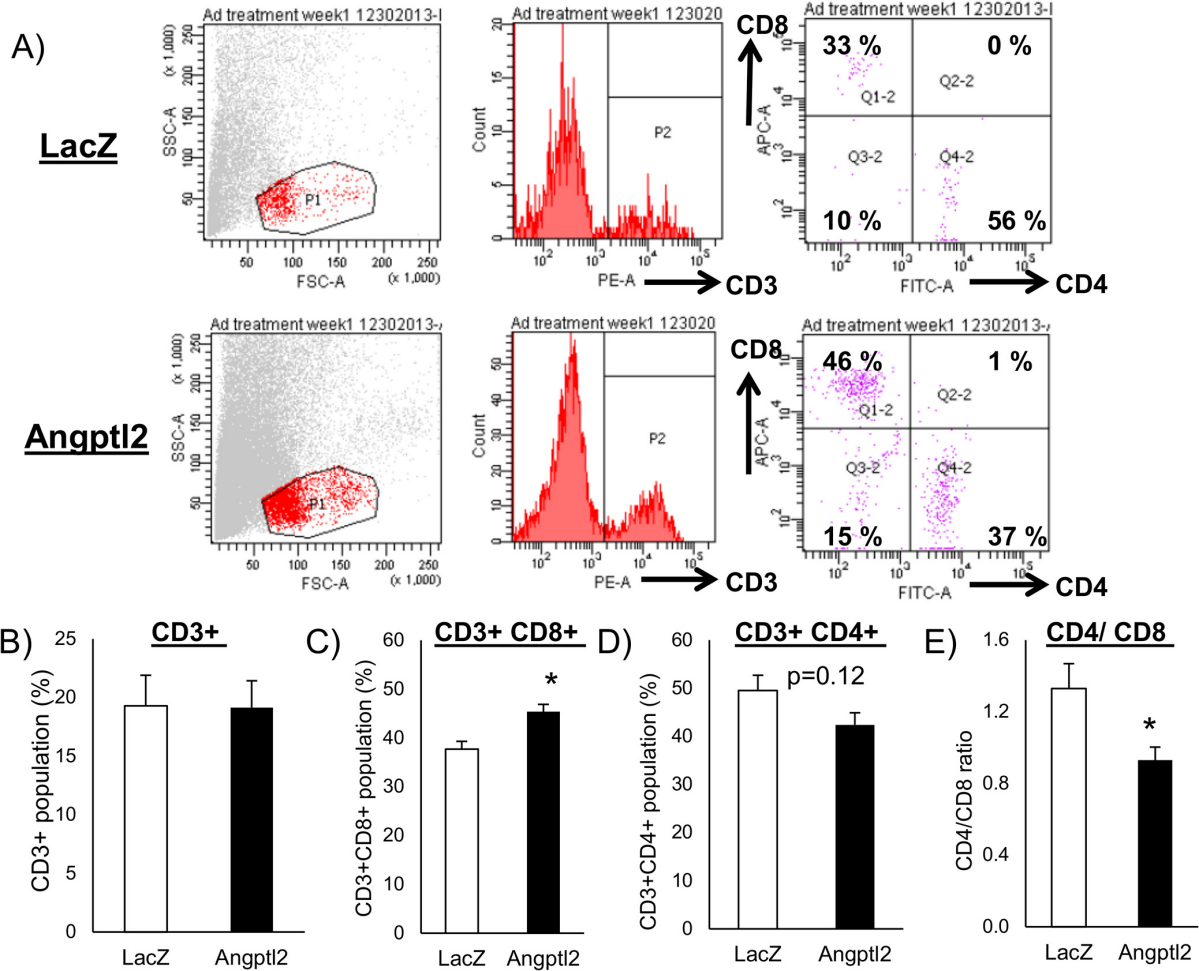
Enforced expression of human ANGPTL2 induced accumulation of CD8 Positive T cells in db/db mice. A, Stromal vascular fraction (SVF) were isolated from the epididymal fat pad then stained with CD3, CD4, and CD8 antibodies and analyzed by FACS. B, CD3+ population. C, CD3+CD8+ population. D, CD3+CD4+ population. E, CD4/CD8 ratio (B-E: n = 4). Data are mean ± SEM, *: P<0.05 compared with LacZ group.

T lymphocytes interact with macrophages and regulate the inflammatory response. Nishimura et al. revealed that CD8 positive T cells play a pivotal role on macrophage recruitment and adipose tissue inflammation [29]. To assess how ANGPTL2 participates in this process, we examined macrophage and T cell populations in stromal vascular fractions at two time-points: one week and two week after the initiation of Ad-ANGPTL2 treatment. Comparing CD3 positive fraction between Ad-LacZ and Ad-ANGPTL2 mice at one week, there was no difference between two groups (Fig 4A and 4B). CD4+ and CD8+ T cells were then analyzed. ANGPTL2 enforced expression increased CD8+ and decreased CD4+ cells (Fig 4C and 4D). Overall, ANGPTL2 enforced expression shifted CD4/CD8 balance among CD3+ T cells toward the dominance of CD8+ cells (Fig 4E). This shift of CD4/CD8 balance was only observed at one week of adenovirus treatment (S2 Fig).

### ANGPTL2 induced pro-inflammatory response in vitro

**Fig 5:**
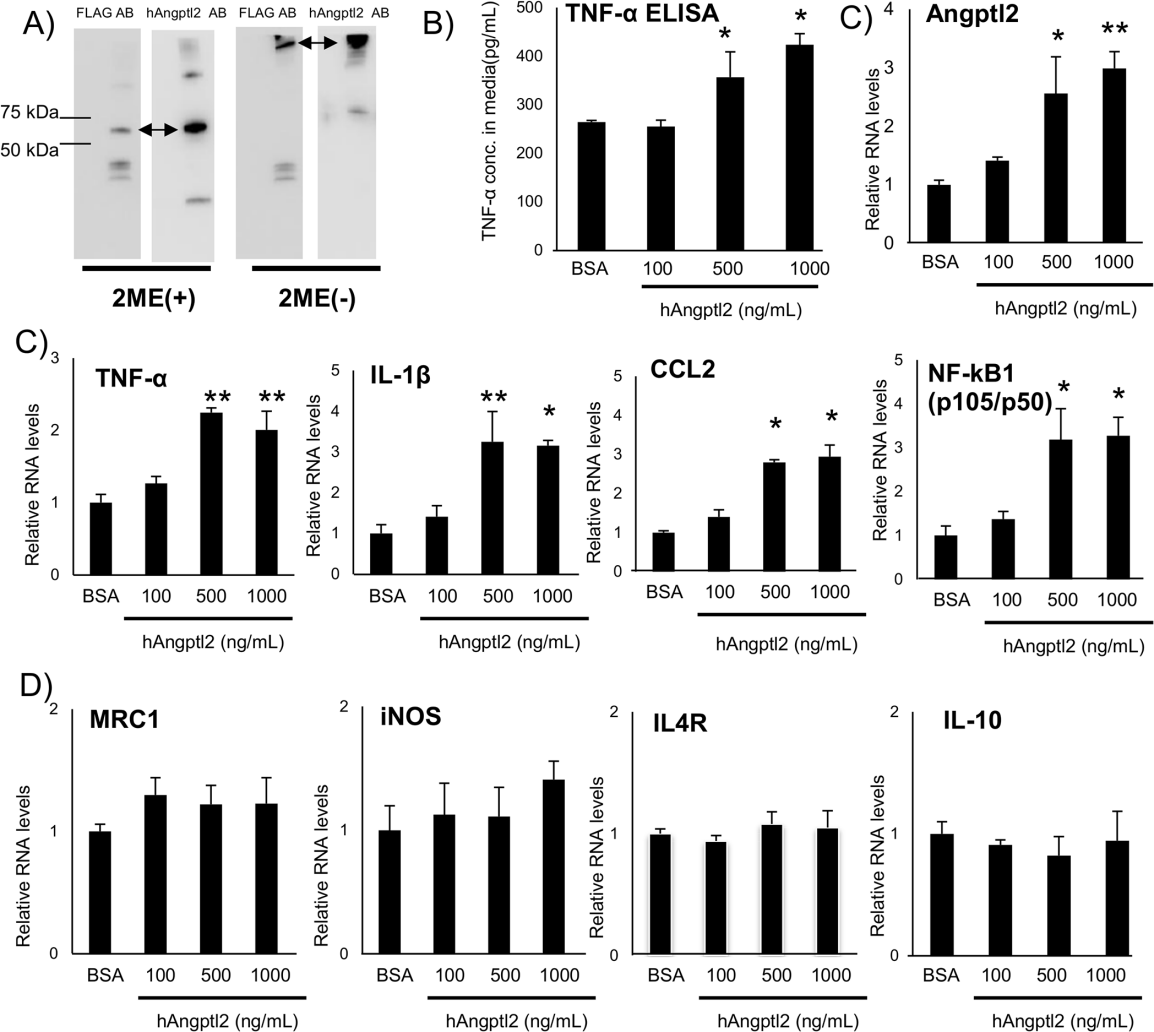
ANGPTL2 induced pro-Inflammatory responses *in vitro*. A, Western blotting analysis of FLAG fusion ANGPTL2 protein with anti-FLAG and anti-human Angptl2 antibody (Left: with 2-mercaptoethanol (2-ME), Right: without 2-ME). B, TNF-α concentration in rANGPTL2-treated THP-1 conditioned media (72 hr treatment, n = 3). C, Quantitative RT-PCR of mRNAs encoding Angptl2 and pro-inflammatory related genes in THP-1 cells (24 hr treatment, n = 3). D, Quantitative RT-PCR of mRNAs encoding M2 macrophage markers in THP-1 cells (24 hr treatment, n = 3). Data are mean ± SEM, **: P<0.01, *: P<0.05 compared with BSA group.

To further provide mechanistic evidence, we prepared recombinant C-terminal FLAG fusion human ANGPTL2 (rANGPTL2) and tested its function of ANGPTL2 in vitro. Western blot analysis detected FLAG-rANGPTL2 protein (Fig 5A). A band shift in non-reduced condition suggests that rANGPTL2 formed multimers consistent with a previous report [23]. rANGPTL2 increased TNF-α production in the human macrophage-like cell line THP-1 (Fig 5B) in a concentration dependent manner. rANGPTL2 treatment increased the expression of pro-inflammatory genes, typical of M1 macrophages, such as TNF-α, IL-1β, CCL2, and NF-κB (p105/p50) in THP-1 cells (Fig 5C). Interestingly, rANGPTL2 treatment also increased human ANGPTL2 expression, probably in an autocrine manner (Fig 5C). In the previous reports, ANGPTL2 activates migration and inflammatory response in EC [15, 19, 20]. We also observed rANGPTL2 treatment induced cell adhesion and inflammatory response in human umbilical vein endothelial cells (HUVEC) (S3 Fig). Collectively, rANGPTL2 induced inflammatory response in macrophage-like THP-1 and endothelial cells. ANGPTL2 induced pro-inflammatory response in THP-1 cells consistent with an increase in larger M1 macrophage subpopulation in adipose tissue in vivo.

### ANGPTL2 increased lipid accumulation in mouse liver

**Fig 6:**
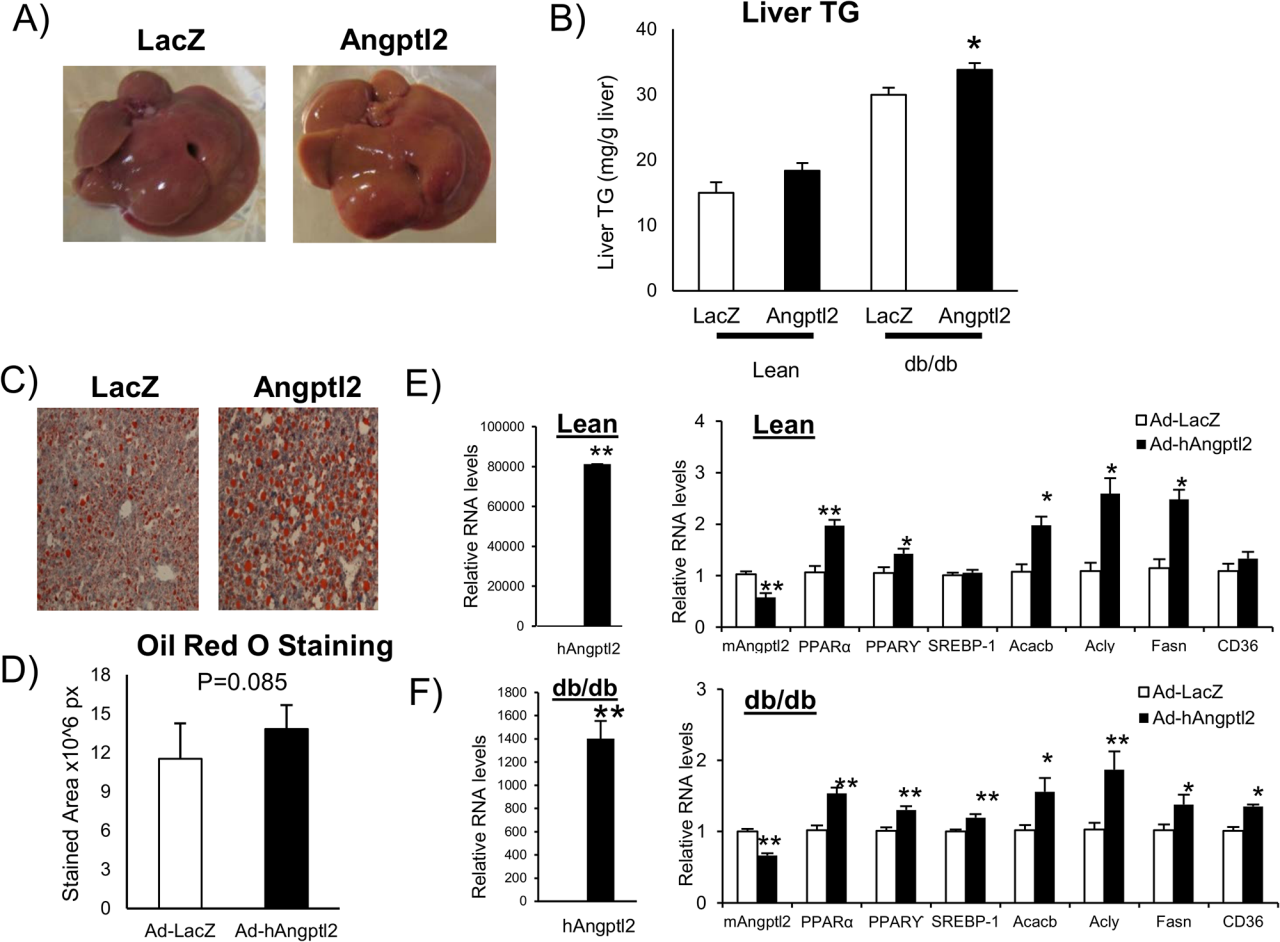
ANGPTL2 enhanced hepatic lipid accumulation in mice. A, Representative images of the liver (Left: LacZ, Right: Angptl2, db/db mice). B, Liver triglyceride levels (Left: Lean mice, Right: db/db mice, n = 7–8 animals per group). C, Representative images of oil red O staining (Left: LacZ, Right: Angptl2, db/db mice). D, Oil red O staining area (n = 7–8, db/db mice). E, Quantitative RT-PCR of mRNAs encoding genes related to fatty acid metabolism in the liver of lean mice (n = 8) F, Quantitative RT-PCR of mRNAs encoding genes related to fatty acid metabolism in the liver of db/db mice (n = 7–8). Data are mean ± SEM, **: P<0.01, *: P<0.05 compared with LacZ group.

While liver weight did not differ between Ad-ANGPTL2 and Ad-LacZ, the liver of Ad- ANGPTL2 treated mice seemed more whitish than did Ad-LacZ treated mice (Fig 6A). Ad-ANGPTL2 increased hepatic triglyceride content in db/db mice as compared to Ad-LacZ (Fig 6B). Oil red O staining showed that the liver of Ad-ANGPTL2 treated mice tended to have greater stained area (p = 0.085, Fig 6C and 6D). Gene expression analysis was assessed in the liver. Human ANGPTL2 gene expression was markedly induced (Fig 6E and 6F). Ad- ANGPTL2 treatment increased the expression of genes related to fatty acid synthesis and metabolism (PPARα, Acacb, Acly, and Fasn) in both lean and db/db mice (Fig 6E and 6F). Although oil red O stain results did not significantly differ, Ad-ANGPTL2 treatment increased fatty acid synthesis, metabolism related gene expression, and lipid accumulation in the liver and may induce fatty liver in mice.

## Discussion

The present study demonstrates that adenovirus-mediated human ANGPTL2 expression in lean and diabetic db/db mice induced adipose tissue inflammation and reduced glucose tolerance and insulin sensitivity. Enforced expression of ANGPTL2 promoted macrophage accumulation and pro-inflammatory M1 polarization in the adipose tissue. ANGPTL2 treatment also enriched CD8+ T cell population at one week of Ad-ANGPTL2 treatment prior to increased macrophage accumulation at two weeks. rANGPTL2 induced pro-inflammatory gene expression and TNF-α protein production in the macrophage-like cell line THP-1. These findings indicate that ANGPTL2 may play important roles in adipose tissue inflammation and insulin resistance via accumulation and pro-inflammatory activation of macrophages.

A previous report by Tabata et al. demonstrated that ANGPTL2-deficient mice ameliorated adipose tissue inflammation and systemic insulin resistance [15]. The same article also demonstrated that adipose tissue-specific Angptl2 overexpression induced fat inflammation and insulin resistance. A more recent study, however, reported opposing effects that four week of recombinant ANGPTL2 treatment improved insulin resistance in db/db mice [22]. Thus, the functional role of ANGPTL2 on insulin resistance remains obscure. To address this issue, we carried out adenovirus-mediated ANGPTL2 expression in lean and diabetic db/db mice, focusing on the function of ANGPTL2 in macrophages. Our results indicated that ANGPTL2 enforced expression clearly induced adipose tissue inflammation and exacerbated insulin sensitivity, which is generally consistent with the results by Tabata et al [15]. A study by Kitazawa et al. and our study used db/db mice as a model of spontaneous type 2 diabetes, however, leading to different outcomes. Two different conditions in two studies—ages of mice and plasma levels of ANGPTL2—may have caused this discrepancy. The previous and our studies used 8 and 12 week old of db/db mice, respectively. Severity of insulin resistance should vary depending on the age of db/db mice, which may in turn affect the function of ANGPTL2 because adipocytes may expose more pro-inflammatory environment and produce more adipokines, leading to more insulin resistant state under more obese condition.

In our study, plasma concentrations of ANGPTL2 ranged from 15–45 ng/mL (Fig 1C and S1C Fig). But, plasma ANGPTL2 levels in the previous report exceeded 100 ng/mL after 2-hour administration, which swiftly decreased afterwards [22]. Integrin α5β1 and LILRB2 proteins were reported as ANGPTL2 receptors [15, 30]. Deng *et al*. revealed that ANGPTL2 forms high-molecular-weight forms. The same study reported that ligand multimerization is required for activation of LILRB2 signaling and that mouse blood contain multimerized ANGPTL2 [31]. Exceeded 100 ng/mL of ANGPTL2 levels were observed in the previous report condition and it was over two folds as much as our condition. It may have contained more multimerized ANGPTL2, causing the different outcomes. In addition, in our condition, plasma ANGPTL2 level was maintained at Day14 after adenovirus injection (S3 Fig), and mice were constantly exposed by ANGPTL2 protein since Ad-ANGPTL2 had begun hANGPTL2 protein expression. We speculate that this persistent stimulation of ANGPTL2 protein induced chronic inflammation and led worse insulin resistance. The functional difference between monomer and multimerized ANGPTL2 is not still fully understood and further investigations will be required.

We assessed that circadian changes in human and mouse Angptl2 in the liver and epididymal white adipose tissue in both lean and db/db mice. Ad-hANGPTL2 treatment markedly induced expression levels of human ANGPTL2 and tended to decrease mouse Angptl2 expression in both the liver and epididymal white adipose tissue (S4, S5, S6 and S7 Figs). Human and mouse Angptl2 sequences were highly conserved (over 95%) and they might exert similar effects in the mouse bodies. Thus, elevated plasma human ANGPTL2 levels might have suppressed endogenous mouse Angptl2 expression through a negative feedback loop in the liver and epididymal white adipose tissue. Additionally, we measured mRNA levels of the circadian genes Clock, Cry1 and Bmal1. As suggested by the reviewer, Angptl2 gene is known as the circadian gene [22]. Kadomatsu et al. reported that human ANGPTL2 gene was significantly induced by Clock and Bmal1 and markedly attenuated by Cry1 co-expression [32]. Therefore, to respond the reviewer’s suggestion, we examined whether the enforced expression of hANGPTL2 affects these circadian gene expression. Our results suggested that increased hANGPTL2 protein levels had minimal effects on the expression of circadian genes (S4, S5, S6 and S7 Figs). Therefore, we concluded that increased human ANGPTL2 levels induced adipose tissue inflammation and led to insulin resistance, not by affecting the circadian gene expression.

ANGPTL2 is known as a pro-inflammatory factor in keratinocytes, adipose tissue and EC [15, 19, 20]. ANGPTL2 promotes chemotaxis of monocytes and leads to macrophage accumulation [15]. Our data also suggest that ANGPTL2 more directly activates pro-inflammatory responses in macrophages (Fig 5B and 5C). In addition, ANGPTL2 expression was induced in an autocrine manner in THP-1 cells (Fig 5C). A similar phenomenon was observed in HUVEC (S8 Fig). Consistent with previous reports, macrophages and EC also produced ANGPTL2 (Fig 5C and S8 Fig) [19, 20, 33]. Furthermore, we assessed the effects of enforced expression of Ad-ANGPTL2 and Ad-LacZ on Akt phosphorylation in 3T3L1 adipocytes, C2C12 murine myoblasts, and HepG2 hepatocellular carcinoma cells, which did not effect on Akt phosphorylation (S9 Fig). This may indicate that ANGPTL2 does not exert direct effects on the insulin signaling pathway. Instead, ANGPTL2 may indirectly induce insulin resistance by enhancing inflammation.

Inflammation in the adipose tissue is an important factor in the development of insulin resistance. Macrophages accumulate in the white adipose tissue in obesity, leading to insulin resistance [4–10]. Recent studies have implicated that some lymphocytes are closely related to this process of macrophage accumulation in white adipose tissue. Nishimura *et al*. showed that infiltration of CD8-positive T cells precedes and contributes to macrophage accumulation during the development of obesity [29]. When we found that Ad-ANGPTL2 treatment increased adipose tissue macrophages and promoted pro-inflammatory M1 macrophage polarization, we speculated that enforced expression of ANGPTL2 induces CD8-positive T cell infiltration in the white adipose tissue before increased macrophage accumulation occurs. Ad-ANGPTL2 treatment for one week substantially increased CD8-positive T cell population without affecting total CD3-positive T cells (Fig 4A, 4B and 4C). This CD8-positive T cell enrichment had disappeared at 2 weeks when macrophage accumulation and M1 polarization increased (S2 Fig and Fig 3). Further investigations are required to determine how ANGPTL2 promotes CD8-positive cell accumulation and subsequent macrophage accumulation and activation in the adipose tissue.

We also observed that Ad-ANGPTL2 treatment induced lipid accumulation in the liver (Fig 6B), increased the expression of fatty acid synthesis and lipid metabolism related genes (Fig 6E and 6F). The function of ANGPTL2 on lipid metabolism remains unclear, but Angptl2 deficient mice showed less fat accumulation in the liver compared to wild type mice [15]. Our results were consistent with the results in these Angptl2 deficient mice. ANGPTL3 and ANGPTL4, other ANGPTL family proteins, are known to be cleaved into the N-terminal coiled-coil domain and the C-terminal fibrinogen like domain. The truncated N-terminal coiled-coil domain from ANGPTL3 and ANGPTL4 can affect lipid metabolism [34, 35]. Likewise, ANGPTL2 is also processed into the N-terminal coiled-coil domain and the C-terminal fibrinogen like domain [36]. Although the function of these truncated forms of ANGPTL2 were not elucidated, it might have a potency to regulate lipid metabolism.

In conclusion, we propose that ANGPTL2 promotes adipose tissue inflammation as gauged by accumulation and activation of macrophages and T lymphocytes, leading to systemic insulin resistance. Understanding the underlying mechanisms by which ANGPTL2 induces adipose tissue inflammation would provide new insight into therapeutic strategies for cardiometabolic diseases caused by insulin resistance.

## Supporting Information

S1 Fig Ad-ANGPTL2 2 week treatment in mice. A, Scheme of experimental procedure which showing time line of adenovirus injection, glucose/ insulin tolerance test, and analysis in this study. B, Body weight (n = 4 animals per group, in experiment3). C, Plasma ANGPTL2 levels at 1 and 2 week after adenovirus injection (Left: 1 week, Right: 2 week, n = 7–8). D, Plasma Insulin level at 2 week after adenovirus injection (Left: Lean mice, Right: db/db mice, n = 7–8 animals per group). E, Plasma adiponectin level at 2 week after adenovirus injection (Left: Lean mice, Right: db/db mice, n = 7–8 animals per group). F, Food intake (Left: Lean mice, Right: db/db mice). G, Plasma total cholesterol level (Left: Lean mice, Right: db/db mice, n = 7–8 animals per group). H, Plasma triglyceride level (Left: Lean mice, Right: db/db mice, n = 7–8 animals per group).Data are mean ± SEM, **: P<0.01, *: P<0.05 compared with LacZ group.
(TIFF)

S2 Fig Ad-ANGPTL2 treatment did not effect on T Lymphocytes at 2 week. A, Stromal vascular fraction (SVF) were isolated from the epididymal fat pad then stained with CD3, CD4, and CD8 antibodies and analyzed by FACS. B, CD3+ population. C, CD3+CD8+ population. D, CD3+CD4+ population. E, CD4/CD8 ratio (B-E: n = 4). Data are mean ± SEM.
(TIFF)

S3 Fig Temporal plasma ANGPTL2 protein Levels in mice (Day14). A, Temporal plasma ANGPTL2 protein levels in lean mice. B, in db/db mice. C, The enlarged graph of plasma mAngptl2 protein levels in lean mice (LacZ treated). D, in db/db mice (LacZ treated). Data are expressed as means ± S.E.M. (n = 2–3 mice for each time point).
(TIFF)

S4 Fig Temporal gene expression of human and mouse Angptl2 and circadian genes in liver of lean mice (Day14). A, Temporal human ANGPTL2 gene expression. B, mouse Angptl2. C, Clock. D, Cry1. E, Bmal1. Data are expressed as means ± S.E.M. (n = 3 mice for each time point).
(TIFF)

S5 Fig Temporal gene expression of human and mouse Angptl2 and circadian genes in liver of db/db mice (Day14). A, Temporal human ANGPTL2 gene expression. B, mouse Angptl2. C, Clock. D, Cry1. E, Bmal1. Data are expressed as means ± S.E.M. (n = 3 mice for each time point).
(TIFF)

S6 Fig Temporal gene expression of human and mouse Angptl2 and circadian genes in epididymal adipose tissue of lean mice (Day14). A, Temporal human ANGPTL2 gene expression. B, mouse Angptl2. C, Clock. D, Cry1. E, Bmal1. Data are expressed as means ± S.E.M. (n = 3 mice for each time point).
(TIFF)

S7 Fig Temporal gene expression of human and mouse Angptl2 and circadian genes in epididymal adipose tissue of db/db mice (Day14). A, Temporal human ANGPTL2 gene expression. B, mouse Angptl2. C, Clock. D, Cry1. E, Bmal1. Data are expressed as means ± S.E.M. (n = 3 mice for each time point).
(TIFF)

S8 Fig ANGPTL2 Induced Pro-Inflammatory Response in HUVEC. Quantitative RT-PCR of mRNAs encoding Angptl2 and pro-inflammatory related genes in HUVEC (24 hr treatment, n = 3). Data are mean ± SEM,*: P<0.05 compared with BSA group.
(TIFF)

S9 Fig Ad-ANGPTL2 treatment did not effect on Akt phosphorylation in 3T3-L1 adipocytes, C2C12 myotubes, and HepG2 cells. A. Western blot analysis on Phospho and Total Akt under Ad-ANGPTL2 treatment in 3T3-L1 adipocytes. MOI (multiplicity of infection): 1000, Insulin stimulation, 100 nM, 15 min (n = 3, each condition). B. Phospho and Total Akt ELISA under Ad-ANGPTL2 treatment in C2C12 myotubes. MOI (multiplicity of infection): 100 or 1000, Insulin stimulation, 100 nM, 15 min (n = 3, each condition). C. Phospho and Total Akt ELISA under Ad-ANGPTL2 treatment in HepG2 cells. MOI (multiplicity of infection): 1000, Insulin stimulation, 10, 100, and 1000 nM, 15 min (n = 3, each condition).
(TIFF)

S1 Table TaqMan probes used for real time PCR (mouse). (TIFF)

S2 Table TaqMan probes used for real time PCR (human). (TIFF)
