# Supplementary figures and images for: Angiopoietin Like Protein 2 (ANGPTL2) Promotes Adipose Tissue Macrophage and T lymphocyte Accumulation and Leads to Insulin Resistance
Source: PLoS One. 2015 Jul 1;10(7):e0131176. doi: 10.1371/journal.pone.0131176 (PMC4489192; doi:10.1371/journal.pone.0131176)

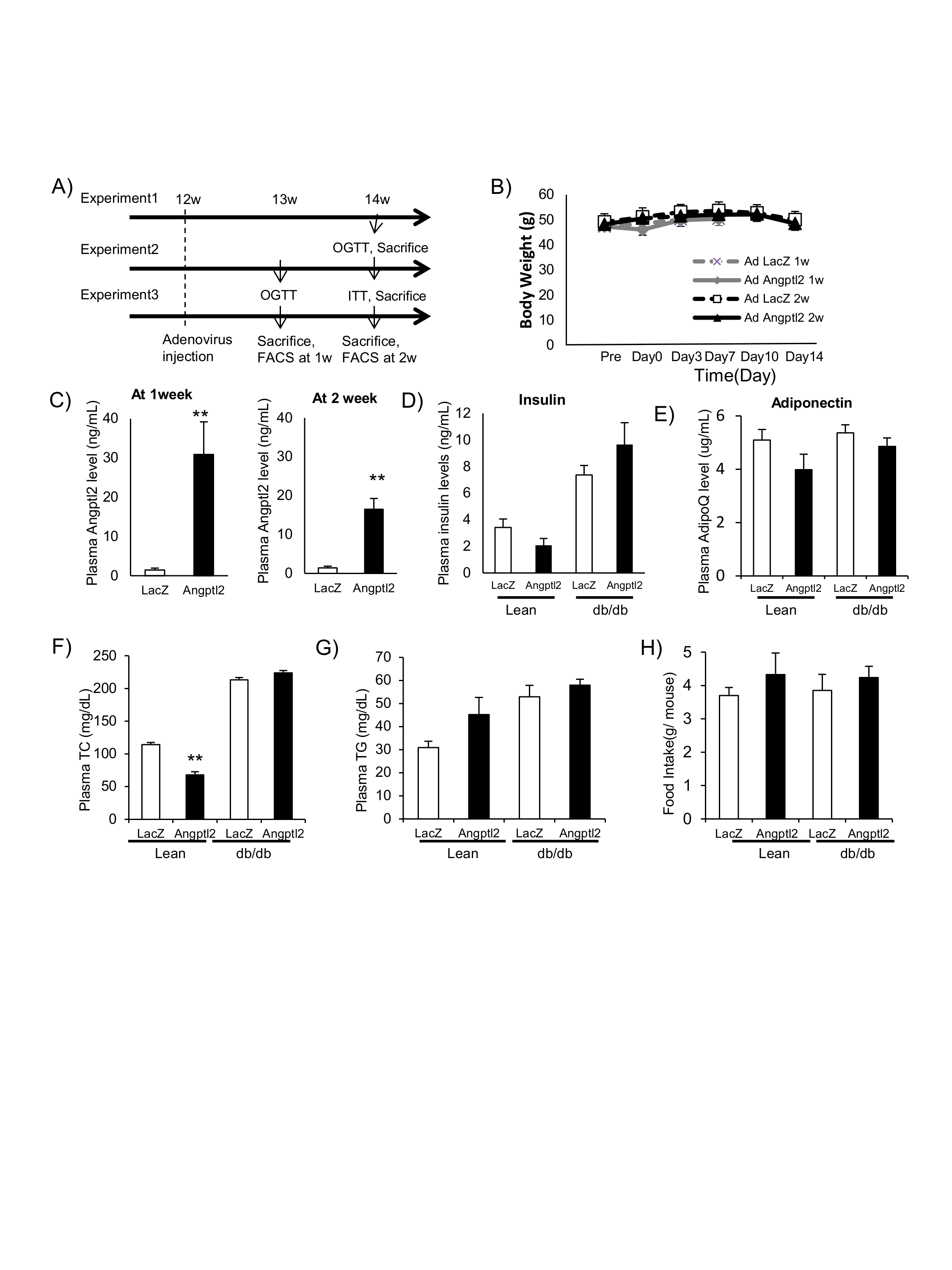

Supplement: S1 Fig — A, Scheme of experimental procedure which showing time line of adenovirus injection, glucose/ insulin tolerance test, and analysis in this study. B, Body weight (n = 4 animals per group, in experiment3). C, Plasma ANGPTL2 levels at 1 and 2 week after adenovirus injection (Left: 1 week, Right: 2 week, n = 7–8). D, Plasma Insulin level at 2 week after adenovirus injection (Left: Lean mice, Right: db/db mice, n = 7–8 animals per group). E, Plasma adiponectin level at 2 week after adenovirus injection (Left: Lean mice, Right: db/db mice, n = 7–8 animals per group). F, Food intake (Left: Lean mice, Right: db/db mice). G, Plasma total cholesterol level (Left: Lean mice, Right: db/db mice, n = 7–8 animals per group). H, Plasma triglyceride level (Left: Lean mice, Right: db/db mice, n = 7–8 animals per group).Data are mean ± SEM, **: P<0.01, *: P<0.05 compared with LacZ group. (TIFF) [file pone.0131176.s001.tiff]

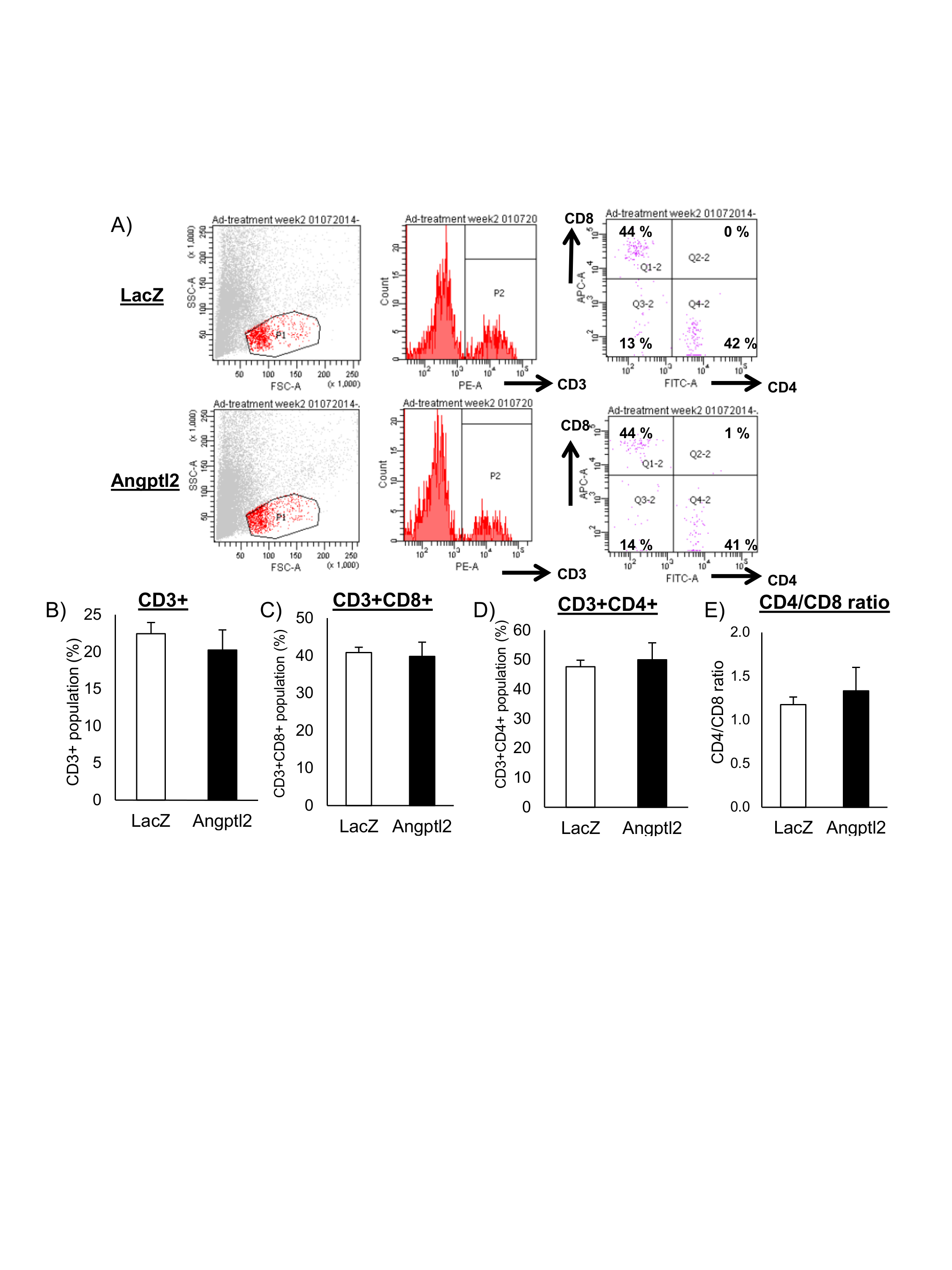

Supplement: S2 Fig — A, Stromal vascular fraction (SVF) were isolated from the epididymal fat pad then stained with CD3, CD4, and CD8 antibodies and analyzed by FACS. B, CD3+ population. C, CD3+CD8+ population. D, CD3+CD4+ population. E, CD4/CD8 ratio (B-E: n = 4). Data are mean ± SEM. (TIFF) [file pone.0131176.s002.tiff]

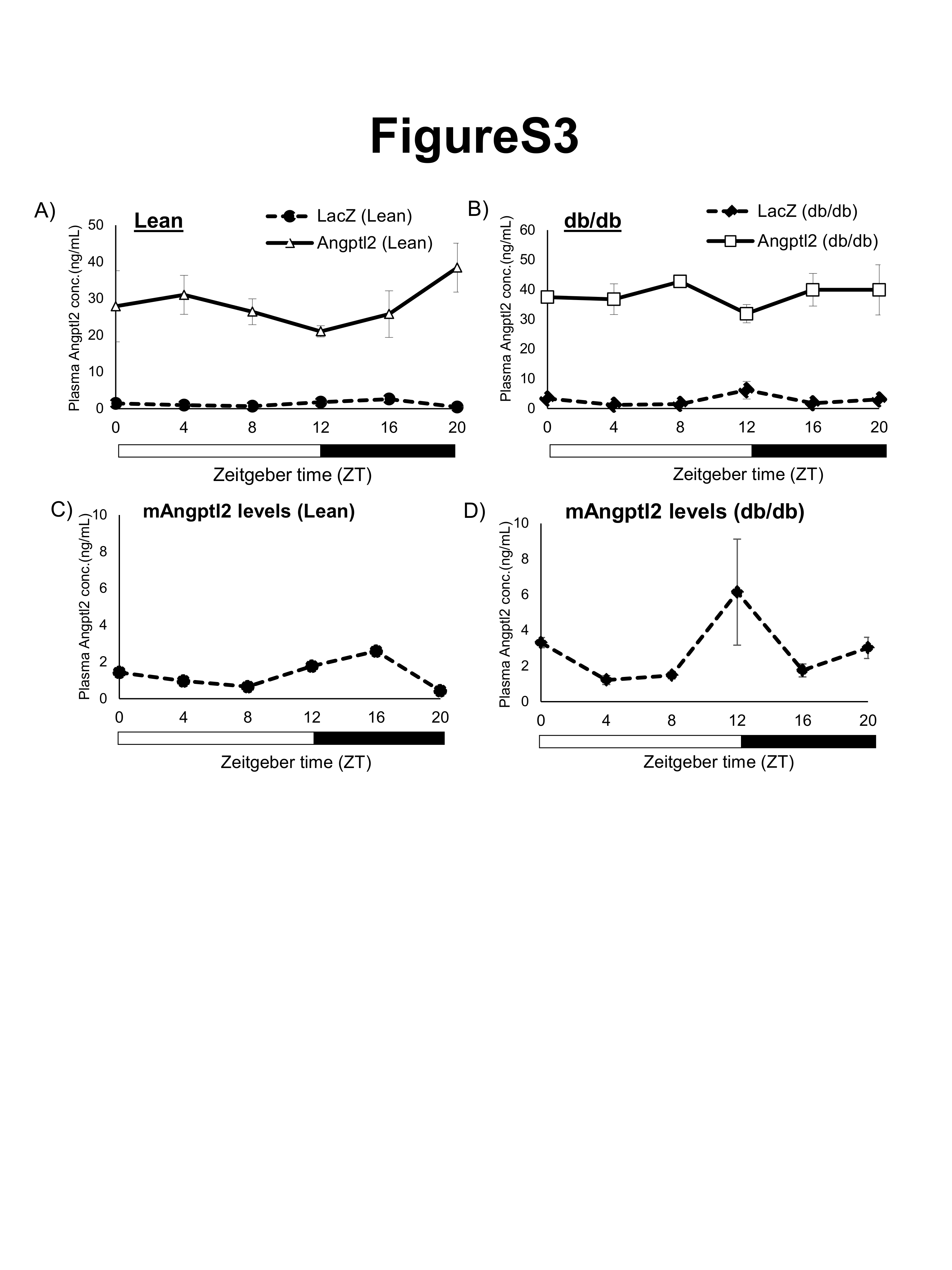

Supplement: S3 Fig — A, Temporal plasma ANGPTL2 protein levels in lean mice. B, in db/db mice. C, The enlarged graph of plasma mAngptl2 protein levels in lean mice (LacZ treated). D, in db/db mice (LacZ treated). Data are expressed as means ± S.E.M. (n = 2–3 mice for each time point). (TIFF) [file pone.0131176.s003.tiff]

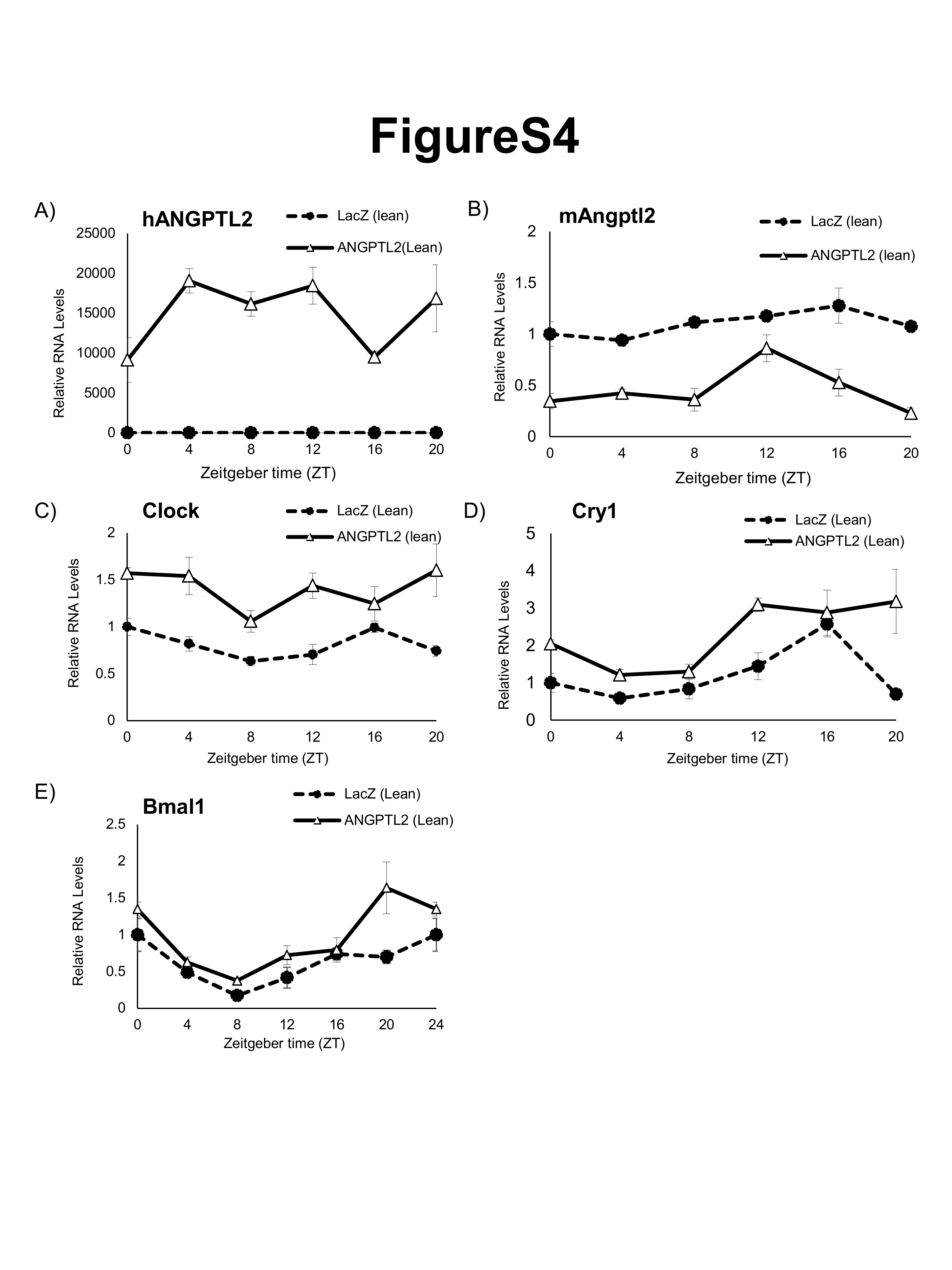

Supplement: S4 Fig — A, Temporal human ANGPTL2 gene expression. B, mouse Angptl2. C, Clock. D, Cry1. E, Bmal1. Data are expressed as means ± S.E.M. (n = 3 mice for each time point). (TIFF) [file pone.0131176.s004.tiff]

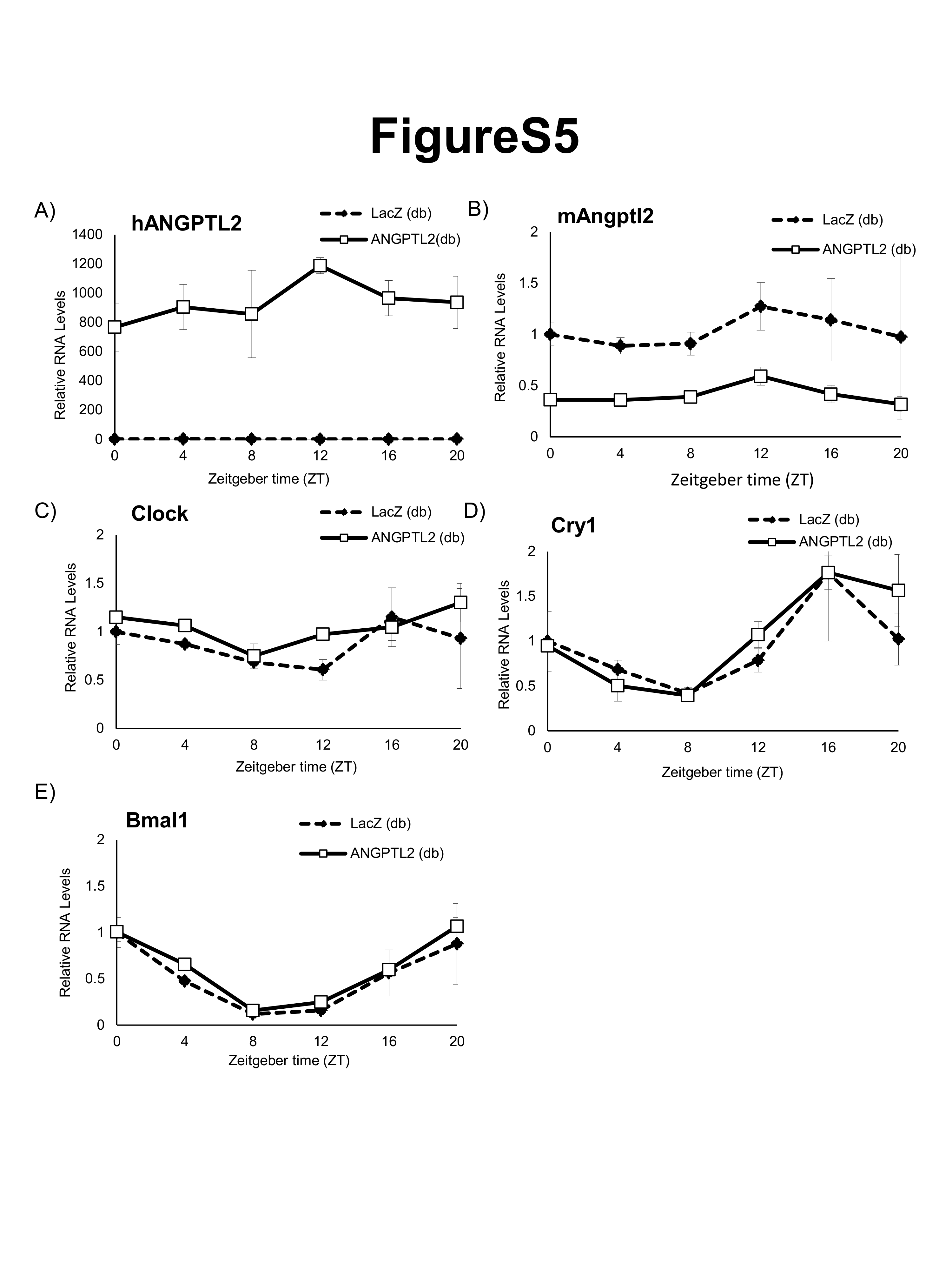

Supplement: S5 Fig — A, Temporal human ANGPTL2 gene expression. B, mouse Angptl2. C, Clock. D, Cry1. E, Bmal1. Data are expressed as means ± S.E.M. (n = 3 mice for each time point). (TIFF) [file pone.0131176.s005.tiff]

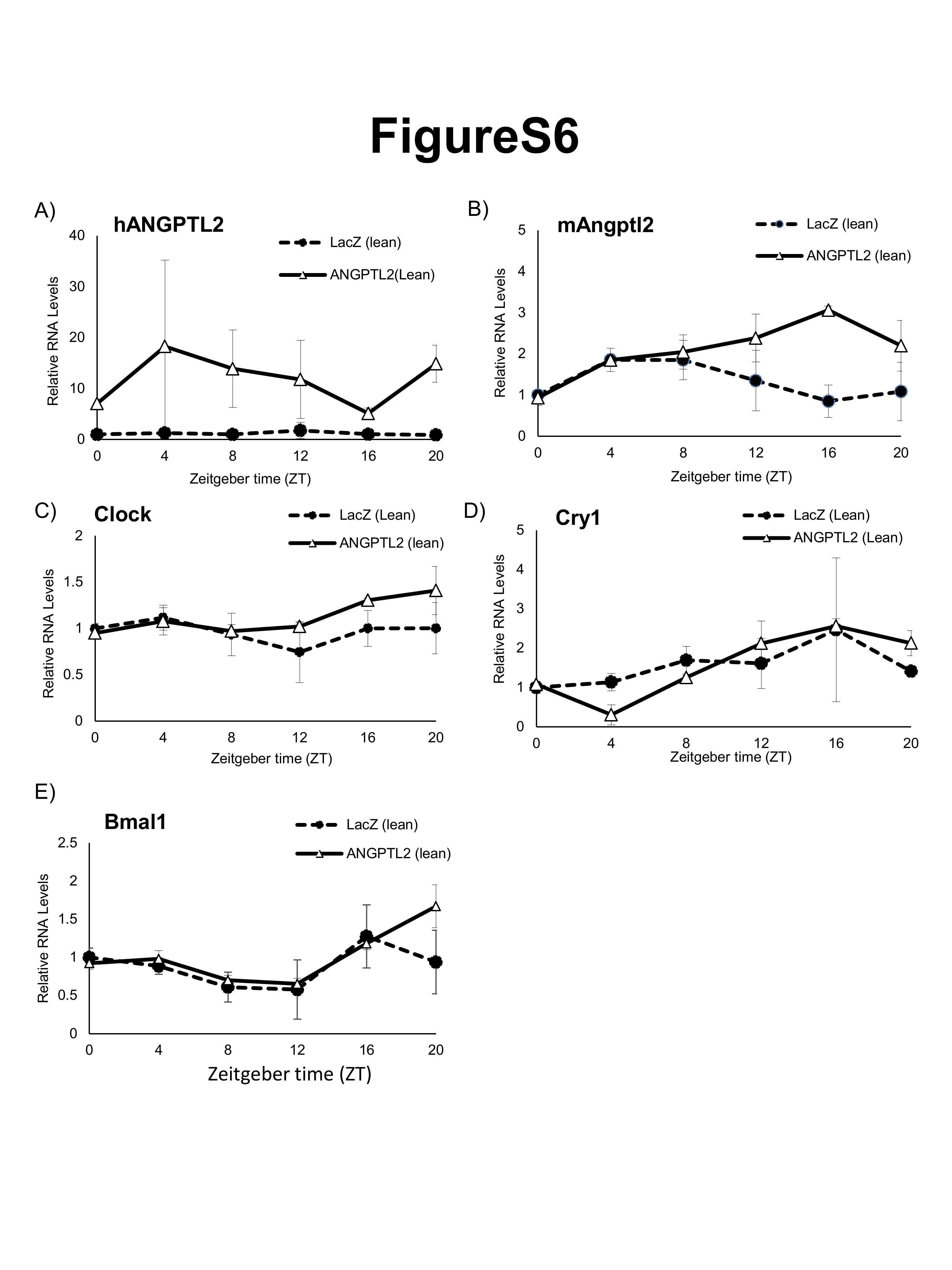

Supplement: S6 Fig — A, Temporal human ANGPTL2 gene expression. B, mouse Angptl2. C, Clock. D, Cry1. E, Bmal1. Data are expressed as means ± S.E.M. (n = 3 mice for each time point). (TIFF) [file pone.0131176.s006.tiff]

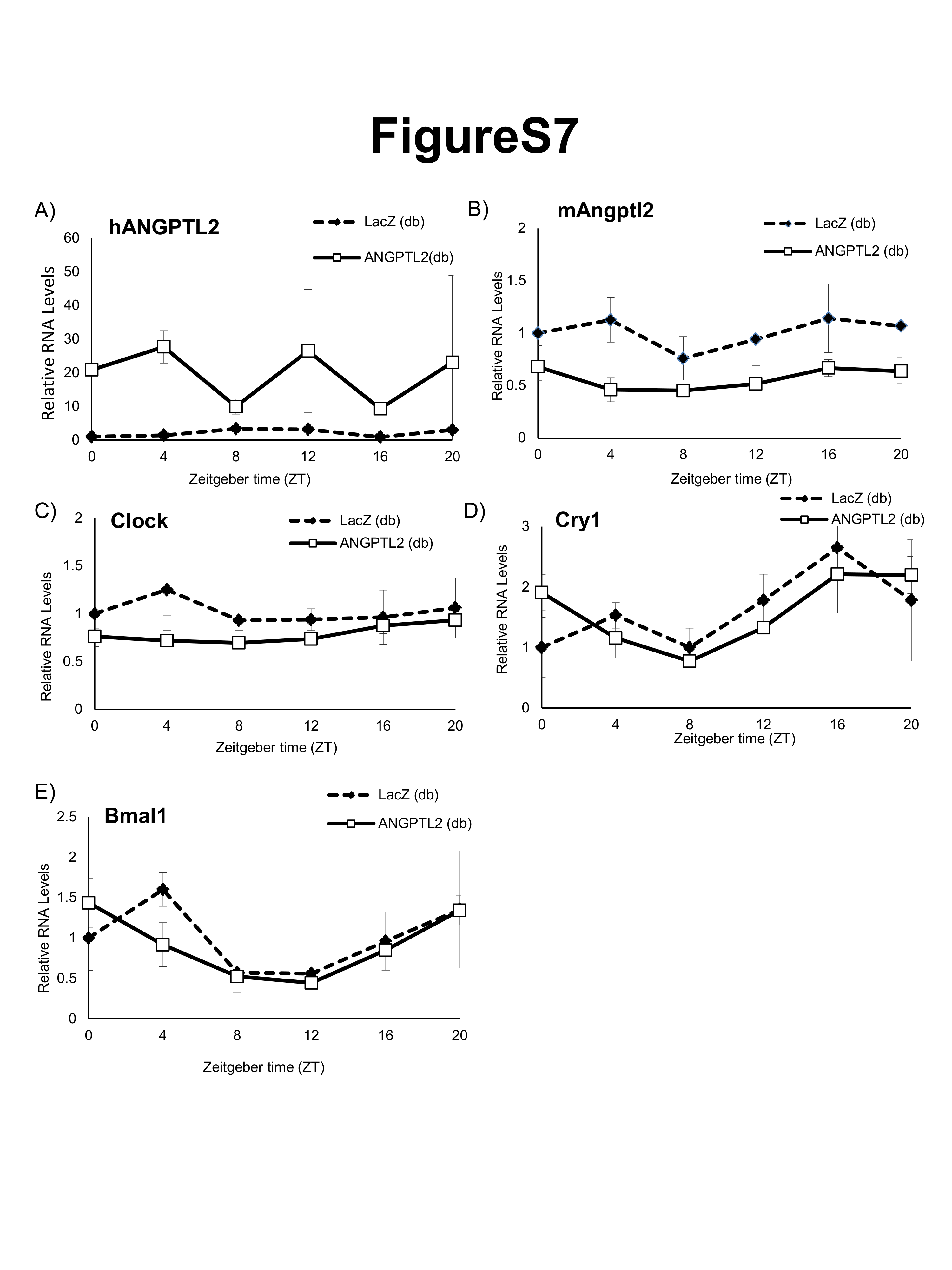

Supplement: S7 Fig — A, Temporal human ANGPTL2 gene expression. B, mouse Angptl2. C, Clock. D, Cry1. E, Bmal1. Data are expressed as means ± S.E.M. (n = 3 mice for each time point). (TIFF) [file pone.0131176.s007.tiff]

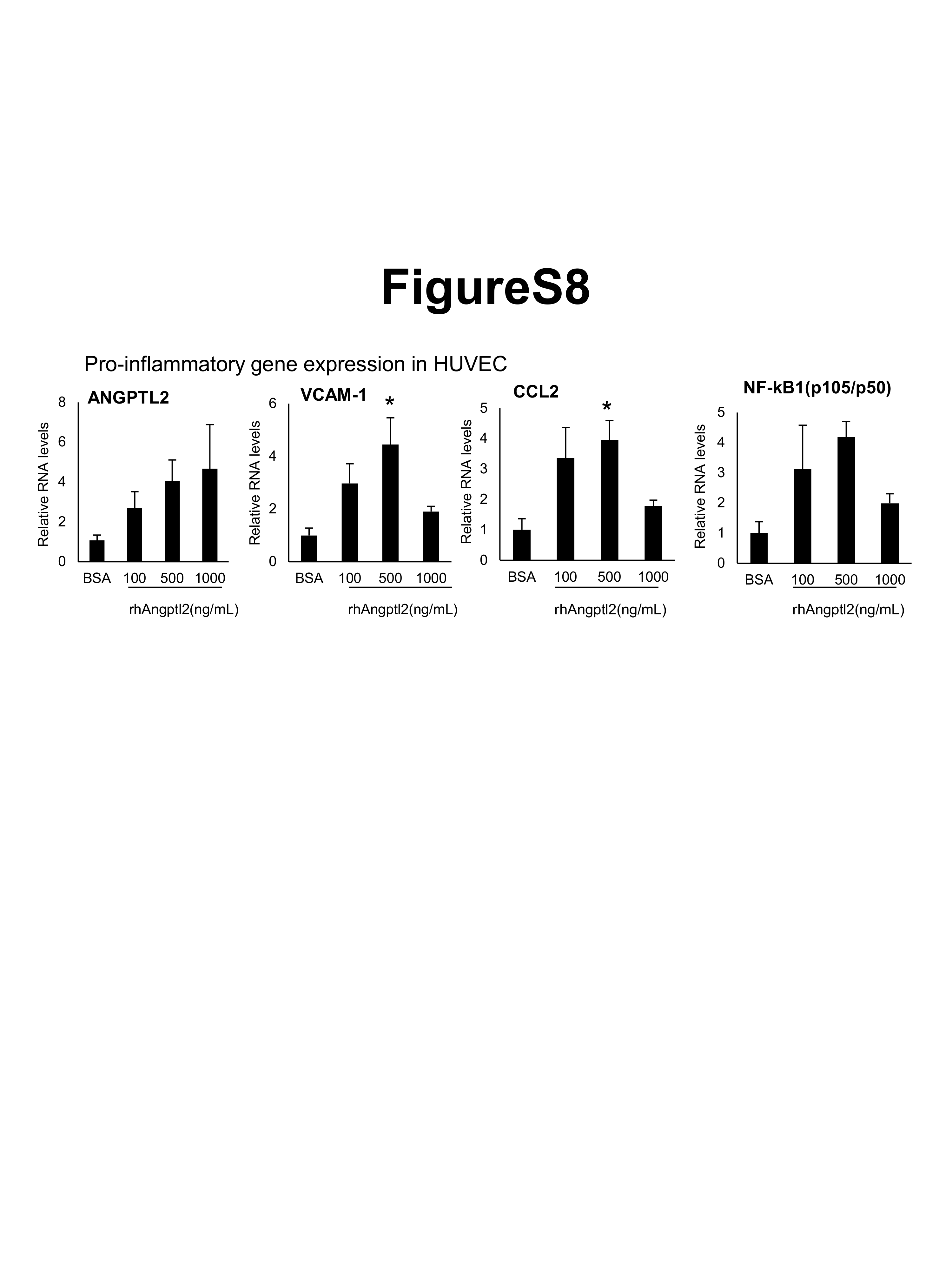

Supplement: S8 Fig — Quantitative RT-PCR of mRNAs encoding Angptl2 and pro-inflammatory related genes in HUVEC (24 hr treatment, n = 3). Data are mean ± SEM,*: P<0.05 compared with BSA group. (TIFF) [file pone.0131176.s008.tiff]

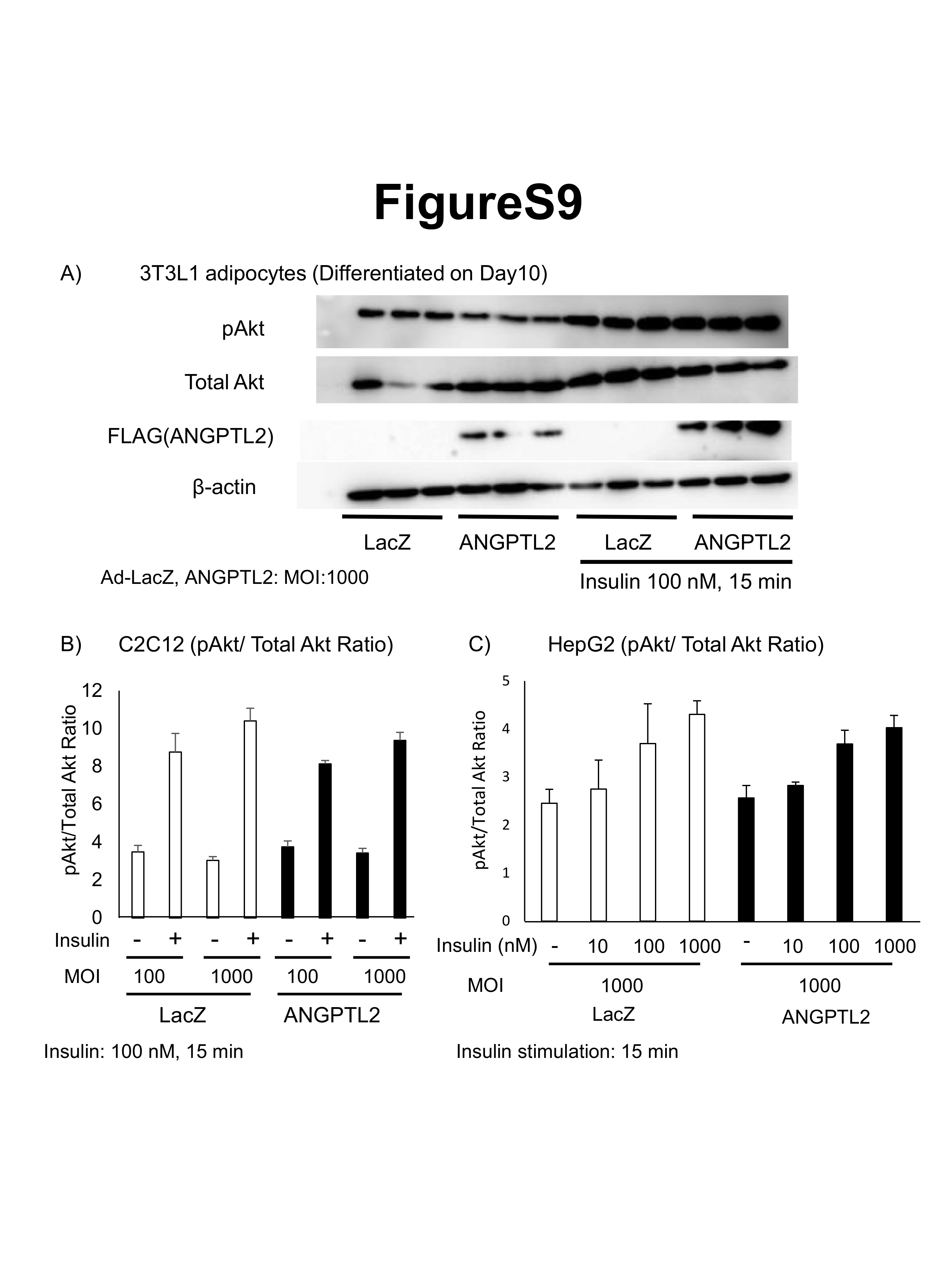

Supplement: S9 Fig — A. Western blot analysis on Phospho and Total Akt under Ad-ANGPTL2 treatment in 3T3-L1 adipocytes. MOI (multiplicity of infection): 1000, Insulin stimulation, 100 nM, 15 min (n = 3, each condition). B. Phospho and Total Akt ELISA under Ad-ANGPTL2 treatment in C2C12 myotubes. MOI (multiplicity of infection): 100 or 1000, Insulin stimulation, 100 nM, 15 min (n = 3, each condition). C. Phospho and Total Akt ELISA under Ad-ANGPTL2 treatment in HepG2 cells. MOI (multiplicity of infection): 1000, Insulin stimulation, 10, 100, and 1000 nM, 15 min (n = 3, each condition). (TIFF) [file pone.0131176.s009.tiff]

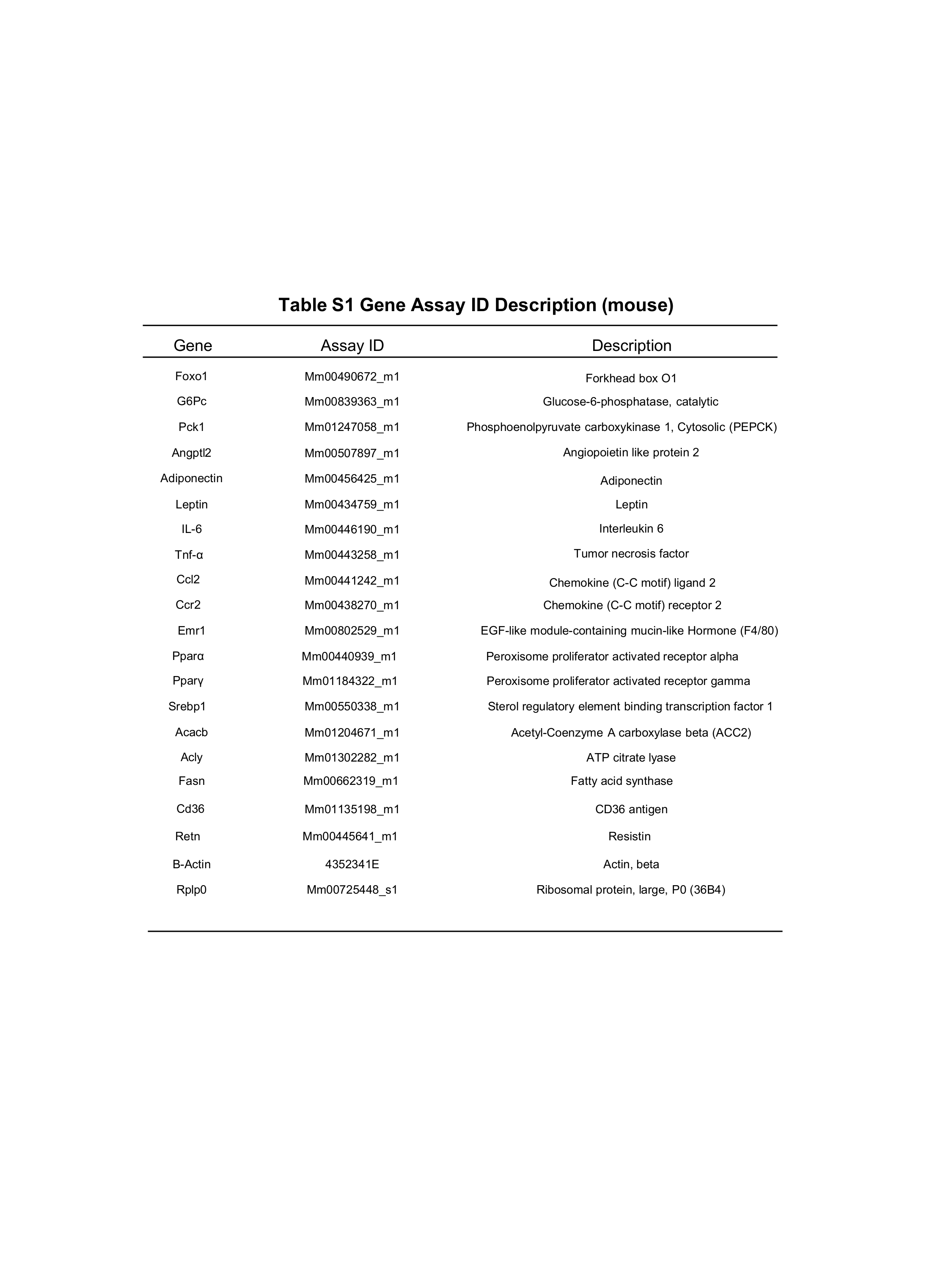

Supplement: S1 Table — (TIFF) [file pone.0131176.s010.tiff]

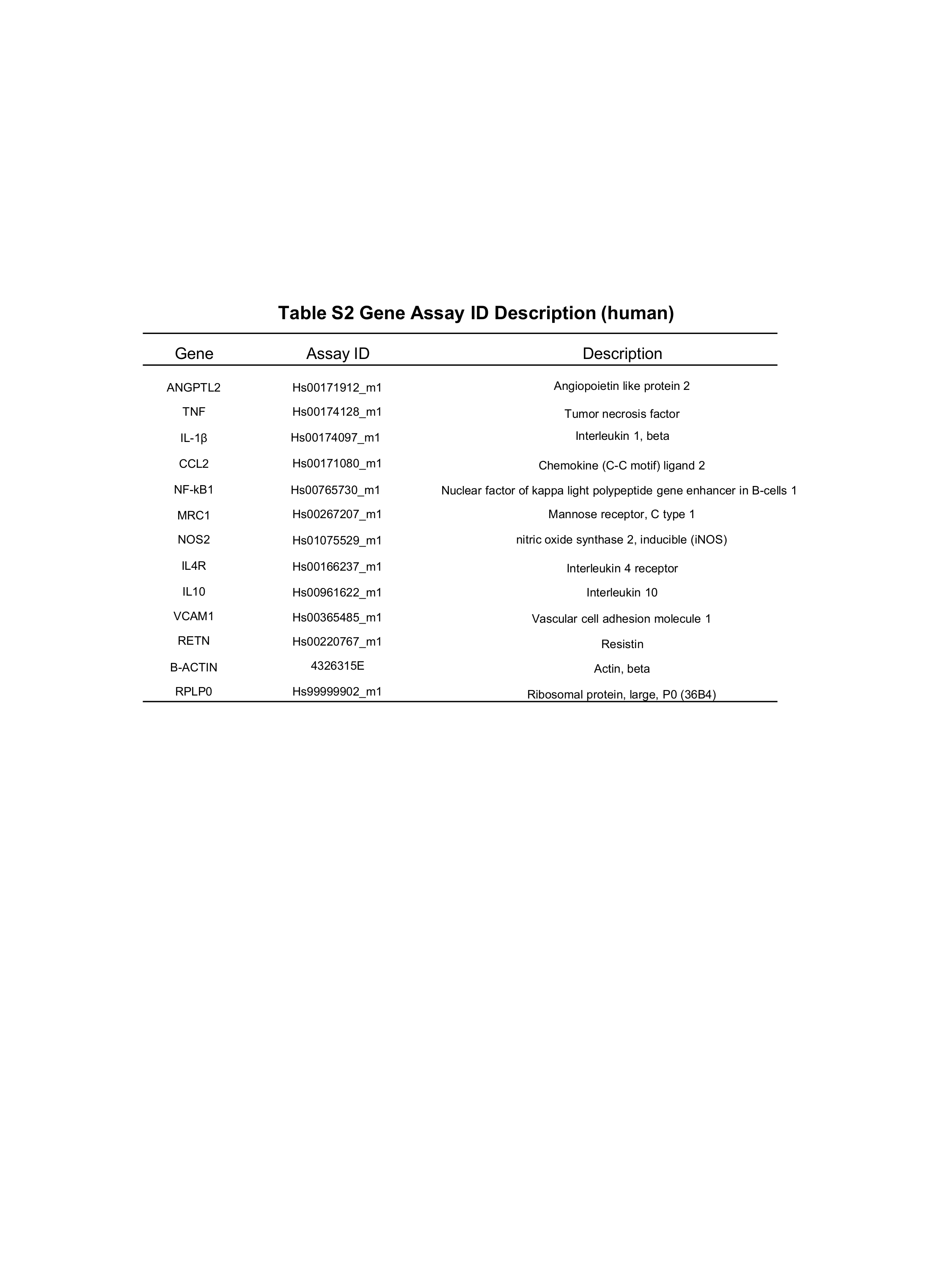

Supplement: S2 Table — (TIFF) [file pone.0131176.s011.tiff]
